# AMPA receptor-mTORC1 signaling activation is required for neuroplastic effects of LY341495 in rat hippocampal neurons

**DOI:** 10.1038/s41598-020-58017-3

**Published:** 2020-01-22

**Authors:** Mi kyoung Seo, Le Thi Hien, Min Kyung Park, Ah Jeong Choi, Dae-Hyun Seog, Seong-Ho Kim, Sung Woo Park, Jung Goo Lee

**Affiliations:** 1 Paik Institute for Clinical Research, Inje University, Busan, 47392 Republic of Korea; 2 Department of Health Science and Technology, Graduate School, Inje University, Busan, 47392 Republic of Korea; 3 Departement of Psychiatry, Dong-eui Hospital, Dongeui University, Busan, 47227 Republic of Korea; 4 Department of Biochemistry, College of Medicine, Inje University, Busan, 47392 Republic of Korea; 5 Department of Internal Medicine, College of Medicine, Haeundae Paik Hospital, Inje University, Busan, 48108 Republic of Korea; 6 Department of Convergence Biomedical Science, College of Medicine, Inje University, Busan, 47392 Republic of Korea; 7 Department of Psychiatry, College of Medicine, Haeundae Paik Hospital, Inje University, Busan, 48108 Republic of Korea

**Keywords:** Molecular neuroscience, Neurotransmitters

## Abstract

The group II metabotropic glutamate 2/3 (mGlu_2/3_) receptor antagonist LY341495 produces antidepressant-like effects by acting on mammalian target of rapamycin complex 1 (mTORC1) signaling and α-amino-3-hydroxy-5-methylisoxazole-4-propionate (AMPA) receptors in rodent. We investigated whether LY341495 affects neuroplasticity via these mechanisms in rat primary hippocampal cultures under conditions of dexamethasone (DEX)-induced neurotoxicity. Ketamine was used for comparison. Hippocampal cultures were treated with LY341495 under conditions of DEX-induced toxicity. Changes in mTORC1-mediated proteins were determined by Western blotting analyses. Changes in dendritic outgrowth and spine density were evaluated via immunostaining. LY341495 significantly prevented DEX-induced decreases in the levels of mTORC1, 4E-BP1, and p70S6K phosphorylation as well as the levels of the synaptic proteins. These effects were blocked by pretreatment with the AMPA receptor inhibitor 2,3-dihydroxy-6-nitro-7sulfamoyl-benzo(f)quinoxaline (NBQX) and the mTORC1 inhibitor rapamycin. LY341495 significantly attenuated DEX-induced decreases in dendritic outgrowth and spine density. Pretreatment with rapamycin and NBQX blocked these effects of LY341495. Further analyses indicted that induction of BDNF expression produced by LY341495 was blocked by pretreatment with NBQX and rapamycin. LY341495 has neuroplastic effects by acting on AMPA receptor-mTORC1 signaling under neurotoxic conditions. Therefore, activation of AMPA receptor and mTORC1 signaling, which enhance neuroplasticity, may be novel targets for new antidepressants.

## Introduction

Depression is a very common mental disorder characterized by depressed mood, decreased interest, decreased cognitive function, and a chronic course with multiple physical symptoms^1^. Antidepressant drugs are the most commonly used method of treating depression^2^. Most antidepressant drugs currently used in clinical practice modulate the function of monoamines, such as serotonin, dopamine, and noradrenaline, in the central nervous system^3,4^.

However, the current research shifts the focus to molecular mechanisms that underlie long-lasting downstream changes in the brain after chronic antidepressant use. Among newly discovered findings regarding the molecular mechanisms of antidepressant drugs, there has been a great deal of research interest in the antidepressant effects of mammalian target of rapamycin (mTOR) signaling activation^5^. A randomized trial reported that ketamine infusion improved the symptoms of depression in patients with treatment-resistant depression^6^. In a systemic review and meta-analysis of patients with depression, a single ketamine infusion was shown to have a rapid antidepressant effect, but the effect lasted from 4 to 7 days^7^. Thus, it has been clinically confirmed that ketamine has an antidepressant effect, but its mechanism of action has not been clearly elucidated. Li *et al*. reported that a sub-anesthetic dose of ketamine activates the mTORC1 signaling pathway, increases the expression of synaptic proteins and synaptogenesis in the prefrontal cortex (PFC) of mice, and exhibits antidepressant effects^8^. Furthermore, for ketamine to have a rapid antidepressant effect, the α-amino-3-hydroxy-5-methyl-4-isoxazolepropionic acid (AMPA) receptor must be stimulated^9,10^. Therefore, activation of mTORC1 and AMPA receptors represent new targets for the development of rapid-acting antidepressants^11^. Although ketamine activates mTORC1 to produce an antidepressant effect, there are obstacles to using this compound as an antidepressant in depressed patients, including the development of dissociative and psychotic symptoms, as well as dependence and drug abuse^12^. Therefore, it is necessary to find drugs that can activate mTORC1 without the side effects of ketamine. Possible alternatives to ketamine include metabotropic glutamate 2/3 (mGlu_2/3_) receptor antagonists. Indeed, mGlu_2/3_ antagonists have been found to have antidepressant effects. According to recent research, LY341495 rapidly increases levels of the phosphorylated and activated forms of ERK and a downstream target of mTORC1, p70S6 kinase, in a concentration- and time-dependent manner in rat primary cortical cultures^13^. Fukumoto *et al*. reported an antidepressant effect in the forced swimming test (FST) when LY341495 was administered to the medial PFC C57BL/6J in male mice. They also found that the antidepressant effect was blocked when the AMPA receptor antagonist NBQX was administered to the mPFC of C57BL/6J male mice^14^. Podkowa *et al*. reported that rats who receive joint administration of ketamine and LY341495 show stronger antidepressant effects in FST, increased mTOR signaling in the PFC and hippocampus, and increased expression of the synaptic proteins GluA1 and PSD95^15^. The mGlu_2/3_ antagonist LY341495 does this by acting on mTORC1 signaling and AMPA receptors^16–18^, and the mechanism may involve effects on the expression of synaptic proteins. However, its effects on the neuroplasticity of primary hippocampal neurons in rats have not been elucidated. In this study, we investigated the effects of this mGlu_2/3_ antagonist on neuroplasticity mediated by mTORC1 signaling and AMPA receptor activity in primary hippocampal neurons of rats. Our reason for working with primary hippocampal neurons was that changes in neuroplasticity in the hippocampus are known to play an important role in the pathophysiology of depression^19^. We also used a dexamethasone-induced neuronal toxic model in this study. Although this was an *in vitro* study, the synthetic corticosteroid dexamethasone is known to increase neuronal death and induce a depression-like phenotype^20,21^. We examined whether LY341495 could promote dendritic outgrowth and spine formation in a toxic environment induced by dexamethasone (DEX). It effects on activation of AMPA receptors and mTORC1 signaling were examined using the AMPA receptor inhibitor 2,3-dihydroxy-6-nitro-7sulfamoyl-benzo(f)quinoxaline (NBQX) and the mTORC1 inhibitor rapamycin. Ketamine was used for comparison.

## Results

### Effects of LY341495 on mTORC1 signaling

To investigate the effects of ketamine and LY341495 on mTORC1 signaling in DEX-treated hippocampal cells, the phosphorylation levels of mTORC1, 4E-BP1, and p70S6K, as well as the expression levels of the synaptic proteins PSD-95 and GluA1, were determined by Western blotting.

**Figure 1:**
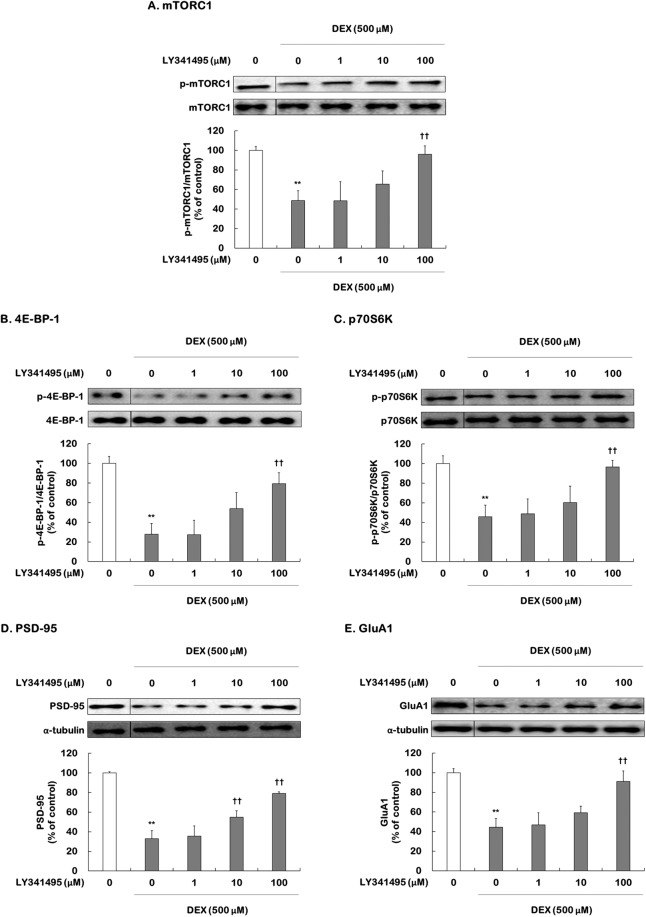
Effects of LY341495 on the levels of mTORC1, 4E-BP1, and p70S6K phosphorylation and PSD-95 and GluA1 expression in DEX-treated hippocampal cells. Cells were treated with LY341495 (1, 10, and 100 μM) or DMSO (non-drug treatment control, final concentration 0.5%) for 4 days with or without DEX (500 μM). The levels of phospho-Ser^2448^-mTORC1 (**A**), phospho-Thr^37/46–^4E-BP1 (**B**), and phospho-Thr^389^-p70S6K (**C**), PSD-95 (**D**), and GluA1 (**E**) were determined by Western blotting. The picture is cropped to eliminate samples of cells from experimental groups not included in this publication. The full picture is provided in the Supplementary File. Values represent the mean ± SEM expressed as a percentage of the value for DMSO-treated, non-DEX-treated cells (control cells). ^**^P < 0.01 vs. DMSO-treated, non-DEX-treated cells; ^††^*P* < 0.01 vs. DMSO-treated, DEX-treated cells.

One-way ANOVA showed significant differences in the levels of mTORC1 (*F*_[4,15]_ = 10.810, *P* < 0.001), 4E-BP (*F*_[4,15]_ = 21.440, *P* < 0.001), p70S6K (*F*_[4,15]_ = 11.910, *P* < 0.001), PSD-95 (*F*_[4,15]_ = 111.900, *P* < 0.001), and GluA1 (*F*_[4,15]_ = 18.240, *P* < 0.001). DEX treatment caused significant decreases in the phosphorylation levels of mTORC1 (48.7% of control, *P* = 0.002), 4E-BP (27.8% of control, *P* < 0.001), and p70S6K (45.7% of control, *P* = 0.001), as well as the expression levels of PSD-95 (33.1% of control, *P* < 0.001) and GluA1 (44.5% of control *P* < 0.001) (Fig. 1). Low (1 μM) to high concentrations (100 μM) of LY341495 increased these phosphorylation levels and synaptic protein expression levels in DEX-treated cells, with 100 μM producing significant increases (phosphor-Ser^2448^-mTORC1: 95.9% of control, *P* = 0.004; phosphor-Thr^37/46–^4E-BP1: 79.2% of control, *P* < 0.001; phosphor-Thr^389^-p70S6K: 96.6% of control, *P* = 0.002; PSD-95: 79.2% of control, *P* < 0.001; GluA1: 91.1% of control, *P* < 0.001). We also found that ketamine (100 μM) increased the levels of mTORC1 signaling-mediated proteins in DEX-treated cells (Supplemental Fig. S1). These results indicate that LY341495 activates mTORC1 signaling, similar to the induction by ketamine, in DEX-treated hippocampal cells.

### Effects of the AMPA receptor inhibitor NBQX on activation of mTORC1 signaling induced by LY341495

The antidepressant actions of ketamine and LY341495 require stimulation of the AMPA receptor^17,22^. In addition, the induction of mTORC1 signaling by ketamine is blocked by AMPA receptor inhibition^17,22^. To determine whether the AMPA receptor is involved in activation of mTORC1 signaling induced by LY341495, DEX-treated hippocampal cells were pretreated with the AMPA receptor inhibitor NBQX and the mTORC1 inhibitor rapamycin for 30 min prior to adding ketamine (100 µM) or LY341495 (100 µM). After a 96-h incubation with ketamine or LY341495, the phospho-Ser^2448^-mTORC1, PSD-95, and GluA1 levels were measured via Western blotting.

**Figure 2:**
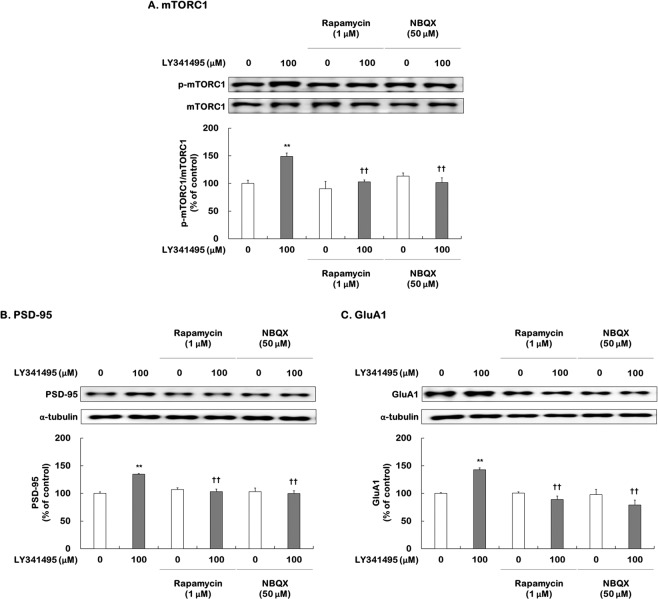
Effects of rapamycin or NBQX on increases in mTORC1 phosphorylation, PSD-95, and GluA1 levels induced by LY341495 in DEX-treated hippocampal cells. Cells were exposed to rapamycin (1 μM, mTORC1 inhibitor) or NBQX (50 μM, AMPA receptor inhibitor) for 30 min prior to adding LY341495 (100 μM) or DMSO (non-drug treatment control, final concentration 0.5%) for 4 days with DEX (500 μM). The levels of phospho-Ser^2448^-mTORC1 (**A**), PSD-95 (**B**), and GluA1 (**C**) were measured using Western blotting. The picture is cropped to eliminate samples of cells from experimental groups not included in this publication. The full picture is provided in the Supplementary File. Values represent the mean ± SEM expressed as a percentage of the value for cells treated with DMSO alone (control cells). ^**^P < 0.01 vs. cells treated with DMSO alone; ^††^*P* < 0.01 vs. cells treated with LY31495 alone.

The results of two-way ANOVA are summarized in Supplemental Table 1. Significant main effects of each drug and each inhibitor on mTORC1 phosphorylation levels and PSD-95 and GluA1 expression levels were found (Supplemental Table 1). Significant interactions were also found (Supplemental Table 1). Rapamycin (1 µM) or NBQX (50 µM) alone did not have a significant effect but completely blocked the ketamine-induced increases in levels of phospho-Ser^2448^-mTORC1, PSD95, and GluA1 (Supplemental Fig. S2). Similarly, LY341495-induced stimulation of mTORC1 phosphorylation (*P* = 0.005) was completely blocked by rapamycin (LY341495 vs. rapamycin + LY341495; 148.9% vs. 102.8%, *P* = 0.008) and NBQX (LY341495 vs. NBQX + LY341495; 148.9% vs. 101.7%, *P* = 0.006) (Fig. 2A). Rapamycin (PSD-95: LY341495 vs. rapamycin + LY341495; 134.6% vs. 103.2%, *P* = 0.001; GluA1: LY341495 vs. rapamycin + LY341495; 142.7% vs. 88.9%, *P* < 0.001) and NBQX (PSD-95: LY341495 vs. NBQX + LY341495; 134.6% vs. 99.6%, *P* = 0.001; GluA1: LY341495 vs. NBQX + LY341495; 142.7% vs. 79.0%, *P* < 0.001) blocked the increases in levels of PSD-95 and GluA1 produced by LY341495 (PSD-95: *P* < 0.001; GluA1: *P* < 0.001) (Fig. 2B,C). Taken together, these results indicate that the stimulation of mTORC1 signaling in response to LY341495 requires activation of the AMPA receptor in DEX-treated hippocampal cells.

### Effects of LY341495 on hippocampal dendritic outgrowth and spine density

Ketamine increases mTORC1 signaling, and this effect induces increased synaptogenesis in the prefrontal cortex^8^. To examine whether LY341495 increases neuroplasticity, ketamine (100 μM) or LY341495 (1, 10, and 100 μM) was added to hippocampal cells cultured with DEX (500 μM) and incubated for 5 days. Total dendritic length and spine formation were measured using antibodies to the dendritic marker MAP-2 and the spine marker phalloidin, respectively.

**Figure 3:**
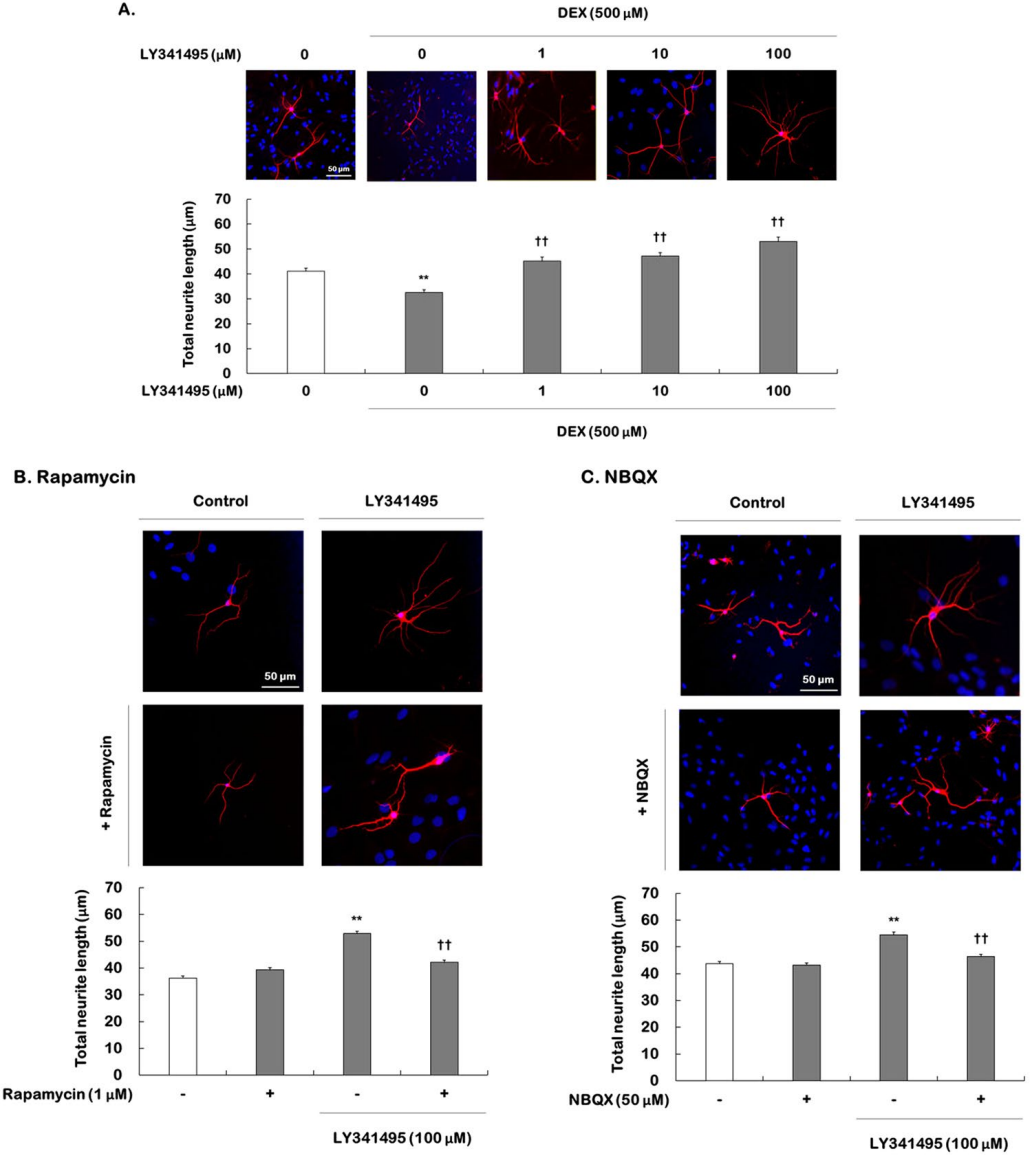
Effects of LY341495 on total dendritic length in DEX-treated hippocampal cells: requirement for mTORC1 signaling and AMPA receptor activation. Cells were treated with LY341495 (1, 10, or 100 μM) or DMSO (non-drug treatment control, final concentration 0.5%) for 5 days with or without DEX (500 μM) (**A**). Cells were exposed to rapamycin (1 μM, **B**) or NBQX (50 μM, C) for 30 min prior to adding LY341495 (100 μM) or DMSO (control) for 5 days with DEX. In total, 400 cells from each group were analyzed. All data are expressed as the mean ± SEM. (**A**) ^*^P < 0.01 vs. DMSO-treated, non-DEX-treated cells (control cells); ^†^*P* < 0.01 vs. DMSO-treated, DEX-treated cells; (**B**,**C**). ^**^P < 0.01 vs. cells treated with DMSO alone; ^††^*P* < 0.01 vs. cells treated with LY31495 alone.

One-way ANOVA showed a significant effect on dendritic outgrowth (*F*_[4,1995]_ = 31.880, *P* < 0.001). DEX treatment significantly reduced total dendritic length compared to control cells (41.1 µm vs. 32.6 µm, respectively, *P* < 0.001) and this reduction was reversed by LY341495 in a concentration-dependent manner (1 μM = 45.2 µm, 10 μM = 47.2 µm, and 100 μM = 53.1 µm, all *P* < 0.001, Fig. 3A). In addition, ketamine at a dose of 100 μM significantly increased total dendritic length in DEX-treated hippocampal cells (Supplemental Fig. S3A). To investigate the roles of mTORC1 signaling and AMPA receptors in the enhancement of dendritic outgrowth induced by LY341495 or ketamine, DEX-treated hippocampal cells were pretreated with rapamycin (1 μM) and NBQX (50 μM) (Fig. 3). Two-way ANOVA revealed significant differences in all scenarios (Supplemental Table 2). Rapamycin and NBQX alone did not affect dendritic outgrowth but inhibited the enhancement of total dendritic length induced by ketamine (Supplemental Fig. S3B,C). Similar to the effects of ketamine, LY341495 (100 μM)-induced increases in total dendritic length (all *P* < 0.001) were completely blocked by rapamycin (LY341495 vs. rapamycin + LY341495: 52.9 µm vs. 42.2 µm, respectively, *P* < 0.001) and NBQX (LY341495 vs. NBQX + LY341495, respectively; 54.5 µm vs. 46.3 µm, respectively, *P* < 0.001).

**Figure 4:**
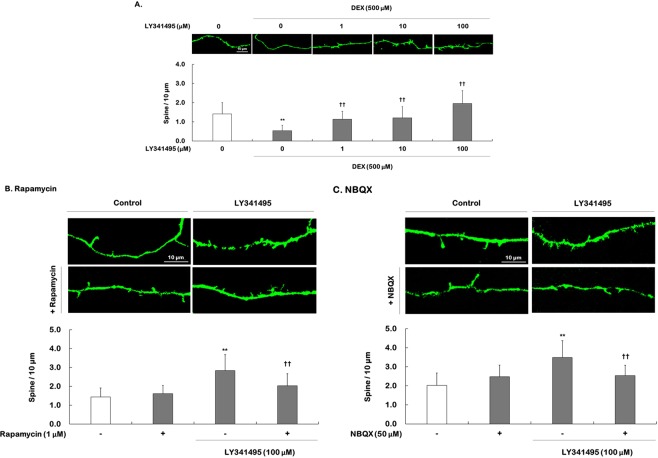
Effects of LY341495 on spine density in DEX-treated hippocampal cells: requirement for mTORC1 signaling and AMPA receptor activation. Cells were treated with LY341495 (1, 10, or 100 μM) or DMSO (non-drug treatment control, final concentration 0.5%) for 5 days with or without DEX (500 μM) (**A**). Cells were exposed to rapamycin (1 μM, **B**) or NBQX (50 μM, **C**) for 30 min prior to adding LY341495 (100 μM) or DMSO for 5 days with DEX. In total, 50–60 dendritic segments from each group were analyzed. All data are expressed as the mean ± SEM. (**A**) ^**^P < 0.01 vs. DMSO-treated, non-DEX-treated cells (control cells); ^††^*P* < 0.01 vs. DMSO-treated, DEX-treated cells; (**B**,**C**) ^**^P < 0.01 vs. cells treated with DMSO alone; ^††^*P* < 0.01 vs. cells treated with LY31495 alone.

Changes similar to those observed in total dendritic length were observed for spine density (*F*_[4,195]_ = 23.550, *P* < 0.001), which was significantly reduced in DEX-treated cells compared to control cells (0.5 vs. 1.4, respectively, *P* < 0.001) (Fig. 4). LY341495 significantly increased dendritic spine density in a concentration-dependent manner in DEX-treated cells (1 μM = 1.1, 10 μM = 1.2, 100 μM = 2.0, all *P* < 0.001). Ketamine at a dose of 100 μM also significantly increased spine density in DEX-treated cells (Supplemental Fig. S4A). The effects of rapamycin on increases in spine density induced by LY341495 or ketamine were examined, and two-way ANOVA revealed significant effects of both drug and drug × rapamycin interactions (Supplemental Table 2). Significant differences were also observed in spine density for ketamine, NBQX, and ketamine × NBQX interactions (Supplemental Table 2). Furthermore, statistical analyses of the effects of NBQX on the LY341495-induced increase in spine density revealed significant effects of LY341495 and LY341495 × NBQX interactions and a trend for the effects of NBQX (*P* = 0.074, Supplemental Table 2). *Post hoc* analyses (Fig. 4) showed that rapamycin and NBQX alone had no effect but inhibited the enhancement of spine density induced by LY341495 (LY341495 vs. rapamycin + LY341495, 2.8 vs. 2.0, respectively, *P* < 0.001; LY341495 vs. NBQX + LY341495, respectively, 3.5 vs. 2.5, *P* < 0.001) or ketamine (Supplemental Fig. S4B,C). These results indicate that the enhancement of dendritic outgrowth and spine density induced by LY341495 require activation of mTORC1 signaling and AMPA receptor.

### Effects of LY341495 on BDNF expression: involvement with AMPA receptor-mTORC1 activation

Increases in spine formation induced by ketamine depend on BDNF release via activation of AMPA receptors and subsequent stimulation of mTORC1 signaling^23,24^. To determine whether LY341495 increases BDNF expression, hippocampal cells cultured with DEX (500 μM) were incubated with ketamine (100 μM) or LY341495 (1, 10, and 100 μM).

**Figure 5:**
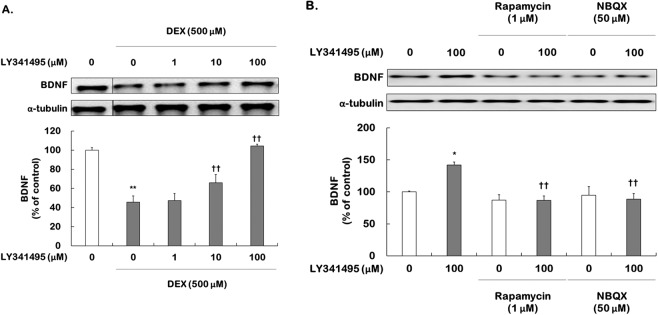
Effects of LY341495 on BDNF expression in DEX-treated hippocampal cells: requirement for mTORC1 signaling and AMPA receptor activation. (**A**) Cells were treated with LY341495 (1, 10, and 100 μM) or DMSO (non-drug treatment control, final concentration 0.5%) for 4 days with or without DEX (500 μM). The levels of BDNF expression were measured using Western blotting. The picture is cropped to eliminate samples of cells from experimental groups not included in this publication. The full picture is provided in the Supplementary File. Values represent the mean ± SEM expressed as a percentage of the value for DMSO-treated, non-DEX-treated cells (control cells). ^*^P < 0.01 vs. DMSO-treated, non-DEX-treated cells; ^†^*P* < 0.01 vs. DMSO-treated, DEX-treated cells. (**B**) Cells were exposed to rapamycin (1 μM, mTORC1 inhibitor) or NBQX (50 μM, AMPA receptor inhibitor) for 30 min prior to adding LY341495 (100 μM) or for 4 days with DEX. Values represent the mean ± SEM expressed as a percentage of the value for cells treated with DMSO alone (control cells). ^*^P < 0.05 vs. cells treated with DMSO alone, ^**^P < 0.01 vs. cells treated with DMSO alone; ^††^*P* < 0.01 vs. cells treated with LY31495 alone.

One-way ANOVA revealed a significant effect on BDNF level (*F*_[4,15]_ = 62.270, *P* < 0.001). DEX treatment significantly decreased the level of BDNF expression compared to control cells (45.7% of control, *P* < 0.002), and LY341495 at concentrations of 10 and 100 μM produced increases in levels of BDNF expression (10 μM = 66.0% of control, *P* = 0.009; 100 μM = 104.4% of control, *P* < 0.001, Fig. 5A), similar to ketamine (Supplemental Fig. S5A). To investigate whether AMPA receptor and mTORC1 signaling activation are required for the upregulation of BDNF induced by LY341495, DEX-treated hippocampal cells were pretreated with NBQX and rapamycin for 30 min prior to adding ketamine (100 µM) or LY341495 (100 µM). After a 96-h incubation, BDNF expression was measured. Two-way ANOVA (Supplementary Table 1) revealed significant main effects in all scenarios. NBQX (50 µM) or rapamycin (1 µM) alone did not affect BDNF expression but each completely blocked the increase in BDNF expression induced by ketamine (Supplemental Fig. S5). Similarly, the effects of LY341495 on BDNF level (*P* = 0.013) were blocked by NBQX (LY341495 vs. NBQX + LY341495, 142.0% vs. 88.8%, respectively, *P* = 0.002) and also rapamycin (LY341495 vs. rapamycin + LY341495, 142.0% vs. 86.7%, respectively, *P* < 0.001) (Fig. 5B).

## Discussion

The mGlu_2/3_ antagonist LY341495 reversed the decreases in mTORC1, 4E-BP, and p70S6K phosphorylation and the decreases in synaptic proteins induced by DEX treatment in primary hippocampal neurons of rats. These effects were blocked by pretreatment of the cells with the mTORC1 inhibitor rapamycin and AMPA receptor inhibitor NBQX. In addition, LY341495 attenuated the decreases in dendritic outgrowth and spine density induced by DEX, and pretreatment with rapamycin and NBQX blocked these effects. LY341495 also increased BDNF expression, and this effect was blocked by rapamycin and NBQX.

Metabotropic glutamate (mGlu) receptors are G protein-coupled modulatory receptors in the glutamate receptor family^25^. There are eight subtypes of the mGlu receptor. The mGlu1 and mGlu2 receptors, which belong to Group I, are mainly excitatory, whereas the mGlu2 and mGlu3 receptors, which belong to Group II, and the mGlu4, mGlu6, mGlu7, and mGlu8 receptors, which belong to Group III, are inhibitory^26^. The following research drugs that bind to orthosteric mGlu_2/3_ receptors have been shown to produce mGlu_2/3_ receptor antagonistic effects: MGS0039, LY341495, and LY3020371^27^. Among these drugs, LY341495 is a highly potent and selective antagonist for mGlu2 and mGlu3 receptors, and several studies have shown that a group II metabotropic glutamate receptor antagonist has antidepressant effects similar to those of ketamine^28^. Bespalov *et al*. reported that intraperitoneal injection of LY341495 reduced the immobility time of mice in the FST^29^. Campo *et al*. reported that pre-administration of LY341495 dose-dependently reduced the immobility time of mice in the FST and that LY341495 also reduced their immobility time in the tail suspension test (TST)^30^. In addition to LY341495, MGS0039 has been shown to produce an increased antidepressant effect in the rat FST and mice TST as the dose increased^31^. Dwyer *et al*. also reported that administration of LY341495 to rats with anhedonia caused by chronic unpredictable stress improved anhedonia rapidly and for a relatively long time^32^. Another study confirmed that the antidepressant effect of LY341495 in the rat FST was blocked by the mTOR inhibitor rapamycin^16^. These findings suggest that mGlu2/3 antagonists may have antidepressant effects.

In addition to the research on antidepressant effects based on these animal models of depression, there have also been studies of the molecular effects of mGlu_2/3_ antagonists. LY341495 increased the phosphorylation levels of mTOR, P70S6K, and 4E-BP1 and the expression of synaptic proteins PSD-95, GluA1, and Synapsin I in the rat prefrontal cortex^16^. LY341495 also increased phosphorylated ERK and phosphorylated S6K levels in primary cortical neurons as a function of dose; BDNF release also increased significantly as a function of time^13^. Chaki *et al*. observed that LY341495 stimulated 5-HT1A receptors in the medial prefrontal cortex (mPFC) of mice to activate PI3K/AKT signaling and induce antidepressant effects. The effect of LY341495 was reduced when rapamycin was administered by mPFC^33^. In addition, the decreased BDNF and PSD-95 expression in the prefrontal cortex, dentate gyrus, and hippocampus CA3 were reversed when MGS00039 was administered to socially-defeated stressed mice^34^. These results suggest that mGlu_2/3_ antagonists can regulate neuroplasticity by activating mTORC1 signaling and increasing expression of synaptic proteins.

In this study, we conducted experiments in a DEX-induced toxicity environment. We confirmed that LY341495 increased mTORC1 signaling in primary hippocampal neurons of rats. DEX is a corticosteroid and has excitotoxic effects on nerve cells. Particularly, exposure to DEX in the prenatal period may be associated with the development of depression. Therefore, we applied the DEX toxicity model in this study^35–37^.

We observed that LY341495 treatment reversed the decreased phosphorylation levels of mTORC1, 4E-BP1 and p70S6K, and the expression of PSD95 and GluA1. Similar results were also observed with ketamine treatment. Li *et al*. observed that ketamine increased the number of synaptic proteins and activated mTORC1 signaling in the PFC of rats^8^. In addition, ketamine has also been shown to activate mTORC1 signaling and increase the expression of synaptic proteins in primary hippocampal neurons of rats^38^. In addition to the activation of mTORC1 signaling, recent studies have shown that ketamine rapidly increased glutamate release and activated AMPA receptors and mTORC1 signaling; the latter, in turn, stimulated BDNF release and Akt activation, facilitating synaptogenesis^39^. Increased BDNF release promotes the synthesis of synaptic proteins, resulting in the rapid antidepressant effect of ketamine. In the present study, we found that the decreases in the expression levels of mTORC1 signaling proteins in the DEX-induced toxic environment were reversed and the decreases in expression levels of BDNF, PSD-95, and GluA1 were also reversed by LY341495 treatment. These results suggest that LY341495, an mGlu_2/3_ antagonist, has a mode of action similar to that of ketamine.

We also evaluated how LY341495 affected AMPA receptors. Several studies have shown that positive allosteric modulators of AMPA receptors have antidepressant effects^27,40^. In one study, rats’ immobility time was significantly decreased by a single LY341495 injection before the FST^17^. In other studies, when NBQX was pre-administered before the FST, the antidepressant effects of LY341495 were dose-dependently decreased, and NBQX alone did not affect the immobility time^17,22^. In another study, NBQX reduced the antidepressant effects of ketamine and LY341495 co-administered in the FST^41^; additionally, pre-administration of LY341495 decreased feeding latency in the novelty-suppressed feeding test, which was blocked by NBQX^42^, and pre-administration of NBQX also blocked increased dopamine release by LY341495 to the basal level in the nucleus accumbens of rats^43^. We observed that the increase in mTORC1 phosphorylation and increases in PSD-95 and GluA1 expression by LY341495 treatment in primary hippocampal neurons were blocked by NBQX. We also found that the increases in mTORC1 phosphorylation and in PSD-95 and GluA1 expression by ketamine were also blocked by NBQX. These results indicate that LY341495 has neuroplastic effects that increase synaptic protein expression by activating the AMPA receptor via a mode of action similar to ketamine^41,44,45^. To the best of our knowledge, this is the first study to examine how LY341495 affects the expression of synaptic proteins by acting on AMPA receptors in rat primary hippocampal neurons.

**Figure 6:**
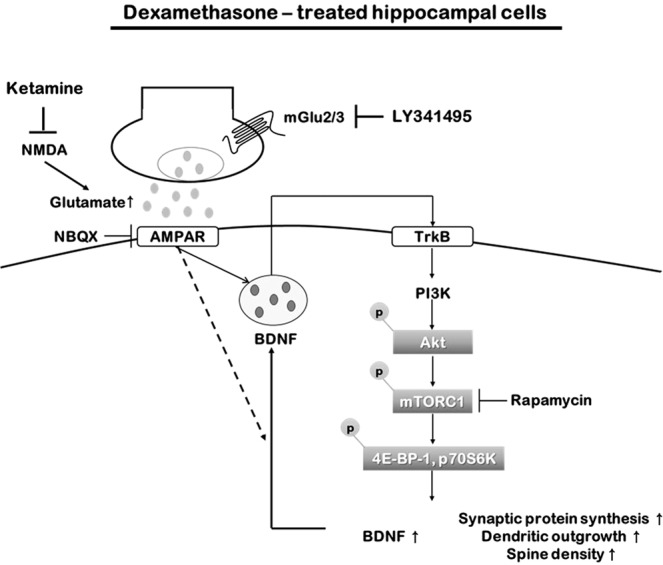
Signaling pathways regulated by LY341495 and ketamine in hippocampal culture under conditions of dexamethasone (DEX)-induced neurotoxicity. Abbreviations: Akt, protein kinase B (PKB); AMPAR, α-amino-3-hydroxy-5-methylisoxazole-4-propionic acid (AMPA) receptor; BDNF, brain-derived neurotrophic factor; mGlu2/3, metabotropic glutamate 2/3; mTORC1, mammalian target of rapamycin complex 1; NBQX, 2,3-dioxo-6-nitro-1,2,3,4-tetrahydrobenzo[f]quinoxaline-7-sulfonamide; NMDA, *N*-methyl-d-aspartate; p70S6K, P70S6 kinase; PI3K, phosphoinositide 3-kinase; TrKB, tropomyosin receptor kinase B receptor; 4E-BP-1, eukaryotic translation initiation factor 4E (eIF4E)-binding protein 1.

Stress affects neuroplasticity, resulting in neuronal changes that lead to chronic depression and altered synaptic density and synapse numbers, which lead to depression^46,47^. Decreased synaptic function and numbers in the dorsolateral prefrontal cortex of depressed patients have also been reported^48^. These results suggest that the depressed brain may show changes in neuroplasticity. In the present study, DEX treatment decreased the BDNF level, total dendritic outgrowth, and spine density of primary hippocampal cells of rats, and both LY341495 and ketamine treatment dose-dependently reversed these effects, confirming that LY341495 affects BDNF level, dendritic outgrowth, and spine density. Our results are similar to those of previous studies^38,49,50^. Zanos *et al*. reported that the ketamine metabolite (2R,6R)-hydroxynorketamine-(2R,6R)-HNK acts on mGlu2 receptor and that combined sub-effective doses of the mGlu_2/3_ receptor antagonist LY341495 and (2R,6R)-HNK have synergistic effects on gamma oscillations and antidepressant-relevant behavioral actions^51^. They also reported that the ketamine metabolite (2R,6R)-hydroxynorketamine-(2R,6R)-HNK increases AMPA-GluA1 receptor expression and activity as well as the level of BDNF and phosphorylation of eEF2K^52^. Lepack *et al*. reported that LY341495 stimulates ERK signaling in primary cortical cultures to promote secretion of BDNF and that this mechanism of action is blocked by pretreatment with the TrkB inhibitor K252a^13^. We also observed that ketamine and LY341495 increased ERK phosphorylation levels (Supplemental Fig. S6). Therefore, although we did not observe antidepressant effects of LY341495 in animal models of depression, LY341495 may have an antidepressant effect with a synaptic mechanism similar to ketamine (Fig. 6).

This study had some limitations. First, LY341495 activated mTORC1 signaling only under toxic conditions. In contrast to our observations, a previous study showed that ketamine promotes mTORC1 signaling in PFC in non-depressed mice^53^. Further investigations, including additional animal model studies, are therefore necessary to clarify this issue. In addition, we used relatively high concentrations of LY341495 in this study. Although LY341495 has strong antagonistic activity against the mGlu_2/3_ receptor, LY341495 does not act only on the mGlu_2/3_ receptor, and LY341495 also has relatively potent antagonist activity and affinity for mGlu7 and mGlu8 receptors^54^. At the concentrations used in the present study, it is possible that LY341495 acted on mGlu7 and mGlu8 receptors as well. Furthermore, it is unclear whether the concentrations of LY341495 used in this study were sufficient for promoting mTORC1 signaling *in vivo*, especially in the human central nervous system. This discrepancy may be due to differences in the conditions of an *in vivo* human brain versus an *in vitro* cell culture. Finally, only 50 µM NBQX was used in this study. Therefore, it is necessary to investigate the effects of NBQX at various other concentrations. More advanced and well-designed studies are necessary to overcome these limitations.

This was the first study to investigate the effects of LY341495 on mTORC1 activation in the primary hippocampal neurons of rats. LY341495 activated the mTORC1 signaling pathway and neuroplastic changes, including increased BDNF expression, dendritic outgrowth, spine density, and synaptic proteins, under conditions of DEX-induced toxicity. These neuroplastic changes were blocked by the mTORC1 inhibitor rapamycin and the AMPA receptor antagonist NBQX. These findings suggest that LY341495 regulates neuroplasticity through AMPA receptors and mTORC1 signaling activation and that LY341495 has a mechanism of action similar to that of ketamine. Therefore, the mechanism of action of the mGlu_2/3_ antagonists may be a suitable target for the development of new antidepressants.

## Methods

### Primary hippocampal culture

All procedures were conducted in accordance with the guidelines of the Institutional Animal Care and Use Committee (IACUC), Inje University, Republic of Korea, and were approved by IACUC at the College of Medicine Inje University (approval no. 2016–044). Primary hippocampal cultures were prepared in a manner similar to that developed by Kaech and Banker^55^ from the brains of Sprague–Dawley (Orient Bio) rat fetuses (embryonic day 17) obtained from pregnant rats. Briefly, hippocampi were dissociated in neurobasal medium (Invitrogen) with trypsin (0.03%; Invitrogen) for 20 min and in neurobasal medium with 1% fetal bovine serum (FBS; Invitrogen), 1% horse serum (Invitrogen), 2% serum-free B27 growth medium (Invitrogen), 0.25% l-glutamine (Invitrogen), and 50 U/mL penicillin–streptomycin (Invitrogen). For Western blotting analyses, cells were plated at 2 × 10^5^ cells per six-well dish. For immunostaining, cells were plated on 18 × 18-mm coverslips in 12-well dishes at a density of 2 × 10^4^ (dendritic outgrowth) and 5 × 10^3^ cells (spine density). Cells were grown at 37 °C and 5% CO_2_ for 10 days.

### Drug treatment

After 10 days of incubation, the cells were cultured with LY341495 (1, 10, 100 µM; Tocris Bioscience) or ketamine (100 µM; Huons) in the presence of DEX (500 µM; Sigma) for 4 days (Western blotting analyses) and 5 days (immunostaining analyses). To study the blocking effects, cells were treated with 50 µM NBQX (Calbiochem) or 1 µM rapamycin (Calbiochem) 30 min prior to LY341495 or ketamine. The culture medium and these drugs were changed every 2 days. A concentration of 500 µM DEX was selected because cell viability was 75–80% at this dose^56^. The concentrations of LY341495 used in this study were based on the observation that these concentrations (1, 10, and 100 µM) lead to concentration-dependent increases in the levels of mTORC1 phosphorylation under DEX-induced toxic conditions; lower concentrations (<1 µM) have no effect on the level of mTORC1 phosphorylation (data not shown). The concentrations of ketamine were based on a previous study that found that this concentration enhances mTORC1 phosphorylation as well as dendritic outgrowth in hippocampal cells^38^.

### Western blotting analyses

Western blotting experiments were performed as described previously^57^. Briefly, hippocampal cells were homogenized in ice-cold lysis buffer (20 mM Tris-HCl, 137 mM NaCl, 10% glycerol, 1% Nonidet™ P-40, 0.1% sodium dodecyl sulfate [SDS], 0.5% sodium deoxycholate, and 2 mM ethylenediaminetetraacetic acid [EDTA]) and one complete protease inhibitor tablet (Roche). The lysates were centrifuged (1000 × *g*, 15 min, 4 °C) and the samples were stored at −80 °C until use. Immunoblotting was performed with one of the primary antibodies (anti-phospho-mTORC1 [Ser2448, #2971], anti-mTORC1 [#2972], anti-phosho-4E-BP-1 [Thr37/46, #2855], anti-4E-BP-1 [#9452], anti-phospho-p70S6K [Thr389, #9205], and anti-p70S6K [#9202], anti-phospho-p44/42 MAPK (ERK1/2) [Thr202/Tyr204, #9101], anti-p44/42 MAPK (ERK1/2) [#4695] [1:1000; Cell Signaling]; anti-PSD-95 [1:1000, AB9634; Millipore]; anti-GluA1 [1:1000, ab109450; Abcam]; anti-BDNF [1:1000; sc-546, Santa Cruz]; anti-α-tubulin [1:2000; T9026, Sigma]) in Tris-buffered saline with Tween-20 (TBS-T) at 4 °C overnight, and then the membranes were washed three times in TBS-T for 10 min. These were incubated for 1 h in TBS-T containing horseradish peroxidase-conjugated secondary antibody (goat-anti-rabbit IgG [1:2000, sc-2004; Santa Cruz] for anti-phospho-mTORC1, anti-mTORC1, anti-phospho-4E-BP-1, anti-4E-BP-1, anti-phospho-p70S6K, anti-p70S6K, anti-PSD-95, anti-GluA1, or anti-BDNF; anti-mouse IgG [1:10000, A4416; Sigma] for anti-α-tubulin) at room temperature. Western blotting analyses were repeated twice per sample for each of the two independent cultures.

### Immunostaining analyses for dendritic outgrowth and spine density

Cells were fixed in 4% paraformaldehyde. For dendritic outgrowth, cells were incubated with anti-microtubule-associated protein (MAP)-2 antibody (1:1000, MAB3418; Millipore). Alexa Fluor® 568 goat anti-mouse IgG (Invitrogen) was used as a secondary antibody, and Hoechst 33258 (Invitrogen) was used for nuclear staining. Stained cells were mounted on cover glasses and observed through a fluorescence microscope (Olympus). To analyze the total dendritic length, five fields were randomly selected from each group, and two independent cultures were performed. All neurons in a given field were counted, including both basal and apical dendrites. Dendritic length was determined as the distance between the edge of the cell body and the tip of the growth cone. Total dendritic length was obtained by summing the lengths of all dendrites from a single neuron and then averaging this measure in each group using the automated image-analysis program MetaMorph (Molecular Devices)^58^. At least 400 cells were analyzed in 10 fields by a researcher blinded to the groups. To determine spine density, cells were incubated with Alexa Fluor® 488 Phalloidin (1:40; Molecular Probes) and mounted for image acquisition. Images were captured at a resolution of 1024 × 1024 pixels, using an LSM 510 META laser-scanning confocal microscope (Carl Zeiss) with a 63 × oil immersion objective lens. To analyze spine density, we differentiated between spines and filopodia by shape and length. Spines were defined as less than 3 µm in length with a round or mushroom shape, and filopodia were defined as between 3 and 10 µm in length with a narrow shape. Ten neurons were randomly selected from each group, and three independent cultures were performed. In each 30 neuron group, two 50 µm dendritic segments per neuron were analyzed (50–60 dendritic segments per group) by a researcher blinded to the groups. To represent the average spine density in a 10 µm dendrite, the spine density of 50 µm dendritic segments was divided by 5.

### Statistical analyses

All statistical analyses were performed using GraphPad Prism software (ver. 7.03; GraphPad Software). Dose studies for ketamine and LY341495 were analyzed by one-way analysis of variance (ANOVA). For blockade experiments, two-way ANOVA was used to assess the main effect of drug (ketamine or LY341495) or inhibitor (rapamycin or NBQX) and the interaction between drug and inhibitor (ketamine × rapamycin and ketamine × NBQX; LY341495 × rapamycin and LY341495 × NBQX). For *post hoc* comparison, Tukey’s multiple-comparison test was carried out. In all analyses, *P* < 0.05 was taken to indicate statistical significance.

## Supplementary information

Supplementary Information.

## Data Availability

The data are available from the corresponding author upon request.
