# Supplementary material for: AMPA receptor-mTORC1 signaling activation is required for neuroplastic effects of LY341495 in rat hippocampal neurons
Source: Sci Rep. 2020 Jan 22;10:993. doi: 10.1038/s41598-020-58017-3 (PMC6976560; doi:10.1038/s41598-020-58017-3)
Supplement: Supplementary file 1 — Supplementary Information. [file 41598_2020_58017_MOESM1_ESM.pdf]

Supplementary Information

AMPA receptor-mTORC1 signaling activation is required for neuroplastic effects of LY341495 in rat hippocampal neurons

Mi kyoung Seo[1], Le Thi Hien[2], Min Kyung Park[3], Ah Jeong Choi[1], Dae-Hyun Seog[4], Seong-Ho Kim[1,5], Sung Woo Park[1,2,6*], and Jung Goo Lee[1,2,7*]

*** Corresponding authors**: Jung Goo Lee, M.D., Ph.D. and Sung Woo Park, Ph.D.

Paik Institute for Clinical Research, Inje University, 75, Bokji-ro, Busanjin-gu, Busan, Republic of Korea. Zip code:47392; Tel: +82 51 890 6749; Fax: +82 51 894 6709; E-mail address: iybihwc@naver.com (J.-G. Lee)/ swpark@inje.ac.kr (S.-W. Park)

List

Page 4; Supplemental Table 1. Summary of two-way analysis of variance results for changes in mTORC1 phosphorylation and PSD-95, GluA1, and BDNF levels

Page 5; Supplemental Table 2. Summary of two-way analysis results for dendritic outgrowth and spine density

Page 6; Effects of ketamine on the phosphorylation levels of mTORC1, 4E-BP1, and p70S6K and PSD-95 and GluA1 expression in DEX-treated hippocampal cells

Page 8; Effects of rapamycin or NBQX on increases in mTORC1 phosphorylation, PSD-95, and GluA1 levels induced by ketamine in DEX-treated hippocampal cells

Page 10; Effects of ketamine on total dendritic length in DEX-treated hippocampal cells: requirement for mTORC1 signaling and AMPA receptor activation

Page 12; Effects of ketamine on spine density in DEX-treated hippocampal cells: requirement for mTORC1 signaling and AMPA receptor activation

Page 13; Effects of ketamine on BDNF expression in DEX-treated hippocampal cells: requirement for mTORC1 signaling and AMPA receptor activation

Page 14; Effects of ketamine and LY341495 on the phosphorylation levels of ERK expression in DEX-treated hippocampal cells

Page 15; Crude gels from Figure 1

Page 19; Crude gels from Figure 2

Page 22; Crude gels from Figure 5

Page 24; Crude gels from Figure S1

Page 28; Crude gels from Figure S2

Page 31; Crude gels from Figure S3

Page 33; Crude gels from Figure S6

Supplemental Table 1. Summary of two-way analysis of variance results for changes in mTORC1 phosphorylation and PSD-95, GluA1, and BDNF levels

	mTORC1		PSD-95		GluA1		BDNF	
	F (1,12)	*P*	*F (1,12)*	*P*	*F (1,12)*	*P*	*F (1,12)*	*P*
Ketamine	35.570	< 0.001	9.252	0.010	10.700	0.007	22.610	< 0.001
Rapamycin	35.130	< 0.001	14.760	0.002	10.600	0.007	52.940	< 0.001
Ketamine × Rapamycin	20.850	< 0.001	11.660	0.005	5.380	0.039	42.740	< 0.001
Ketamine	16.280	0.002	5.804	0.030	7.550	0.018	21.070	< 0.001
NBQX	12.170	0.004	14.280	0.003	9.331	0.010	26.770	< 0.001
Ketamine × NBQX	9.890	0.008	10.930	0.006	5.101	0.043	28.660	< 0.001
LY341495	13.720	0.003	19.290	< 0.001	11.680	0.005	12.140	0.005
Rapamycin	11.410	0.005	10.370	0.007	32.430	< 0.001	33.740	< 0.001
LY341495 × Rapamycin	5.048	0.044	30.940	< 0.001	32.430	< 0.001	13.660	0.003
LY341495	5.812	0.033	11.440	0.005	5.312	0.040	4.824	0.048
NBQX	4.887	0.047	11.440	0.005	34.230	< 0.001	12.670	0.004
LY341495 × NBQX	15.230	0.002	18.360	0.001	27.660	< 0.001	8.725	0.012

BDNF, brain-derived neurotrophic factor; GluA1, AMPA receptor subunit GluR1; mTORC1, mammalian target of rapamycin complex 1; NBQX, 2,3-dioxo-6-nitro-1,2,3,4-tetrahydrobenzo[f]quinoxaline-7-sulfonamide; PSD-95, Post Synaptic Density 95 protein.

Supplemental Table 2. Summary of two-way analysis results for dendritic outgrowth and spine density

	Neurite outgrowth			Spine density		
	df1, df2	***F***	***P***	***df1, df2***	***F***	***P***
Ketamine		75.630	< 0.001		130.000	< 0.001
Rapamycin	1,1196	9.831	0.002	1,196	8.867	0.003
Ketamine × Rapamycin		56.450	< 0.001		18.240	< 0.001
Ketamine		152.900	< 0.001		36.590	< 0.001
NBQX	1,1596	93.860	< 0.001	1,207	6.050	0.015
Ketamine × NBQX		79.710	< 0.001		23.490	< 0.001
LY341495		139.600	< 0.001		52.300	< 0.001
Rapamycin	1,1596	24.230	< 0.001	1,196	6.310	0.013
LY341495 × Rapamycin		67.980	< 0.001		15.490	< 0.001
LY341495		152.900	< 0.001		31.470	< 0.001
NBQX	1,1596	93.860	< 0.001	1,196	3.229	0.074
LY341495 × NBQX		73.710	< 0.001		26.440	< 0.001

NBQX, 2,3-dioxo-6-nitro-1,2,3,4-tetrahydrobenzo[f]quinoxaline-7-sulfonamide.

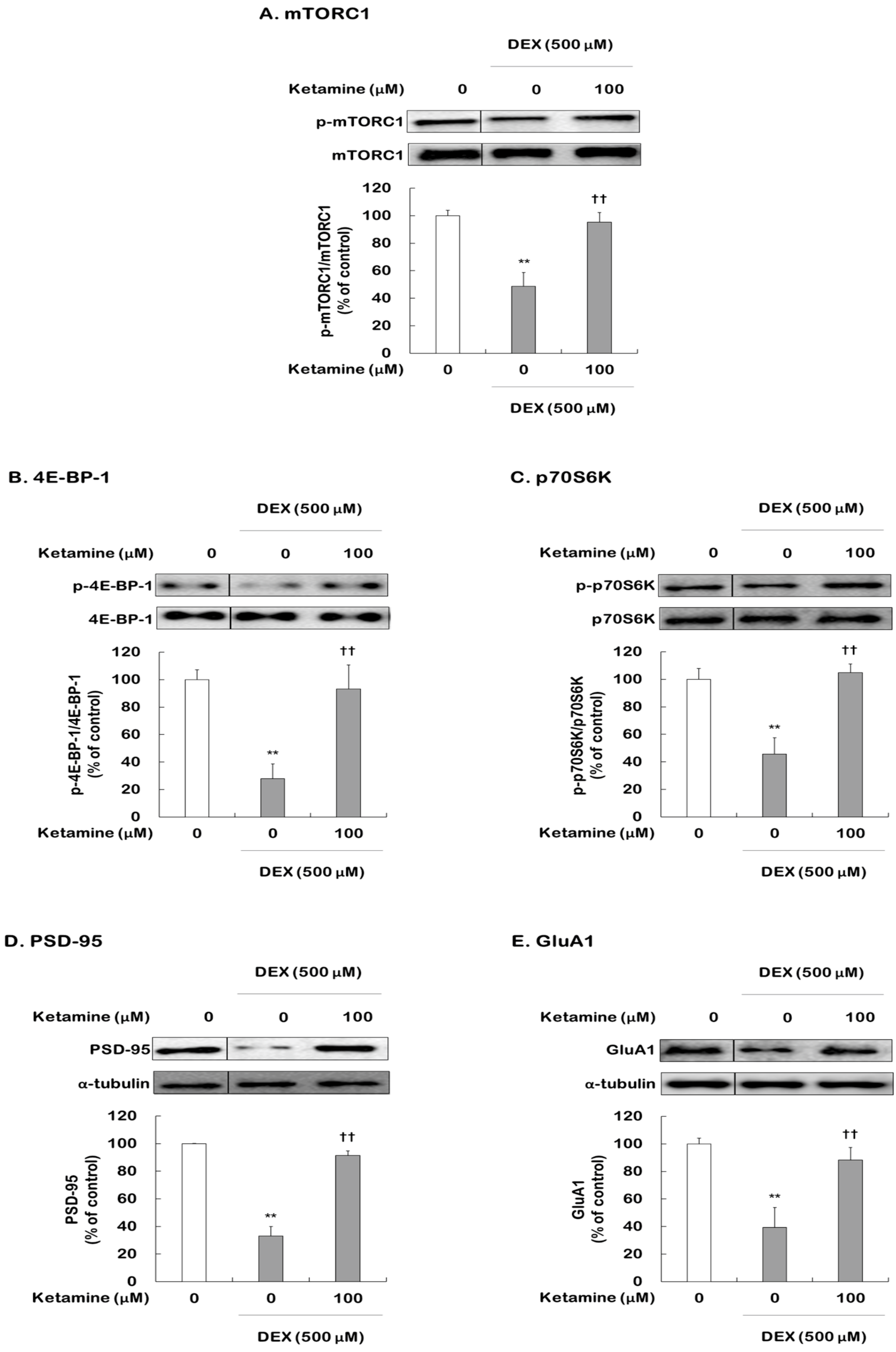
A. mTORC1
DEX (500 μM)
Ketamine (μM) 0 0 100
p-mTORC1
mTORC1
p-mTORC1/mTORC1 (% of control)
120
100
80
60
40
20
0
**
††
Ketamine (μM) 0 0 100
DEX (500 μM)
B. 4E-BP-1
DEX (500 μM)
Ketamine (μM) 0 0 100
p-4E-BP-1
4E-BP-1
p-4E-BP-1/4E-BP-1 (% of control)
120
100
80
60
40
20
0
**
††
Ketamine (μM) 0 0 100
DEX (500 μM)
C. p70S6K
DEX (500 μM)
Ketamine (μM) 0 0 100
p-p70S6K
p70S6K
p-p70S6K/p70S6K (% of control)
120
100
80
60
40
20
0
**
††
Ketamine (μM) 0 0 100
DEX (500 μM)
D. PSD-95
DEX (500 μM)
Ketamine (μM) 0 0 100
PSD-95
α-tubulin
PSD-95 (% of control)
120
100
80
60
40
20
0
**
††
Ketamine (μM) 0 0 100
DEX (500 μM)
E. GluA1
DEX (500 μM)
Ketamine (μM) 0 0 100
GluA1
α-tubulin
GluA1 (% of control)
120
100
80
60
40
20
0
**
††
Ketamine (μM) 0 0 100
DEX (500 μM)

Figure S1. Effects of ketamine on the phosphorylation levels of mTORC1, 4E-BP1, and p70S6K and PSD-95 and GluA1 expression in DEX-treated hippocampal cells.

Cells were treated with ketamine (100 μM) or DMSO (non-drug treatment control, final concentration 0.5%) for 4 days with or without DEX (500 μM). The levels of phospho-Ser^{2448}-mTORC1 (A), phospho-Thr$^{37/46}$–4E-BP1 (B), and phospho-Thr^{389}-p70S6K (C), PSD-95 (D), and GluA1 (E) were measured using Western blotting. The picture is cropped to eliminate samples of cells from experimental groups not included in this publication. The full picture is provided in the Supplementary file. Values represent the mean ± SEM expressed as a percentage of the value for DMSO-treated, non-DEX-treated cells (control cells). $^{**}P < 0.01$ vs. DMSO-treated, non-DEX-treated cells; $^{\dagger\dagger}P < 0.01$ vs. DMSO-treated, DEX-treated cells.

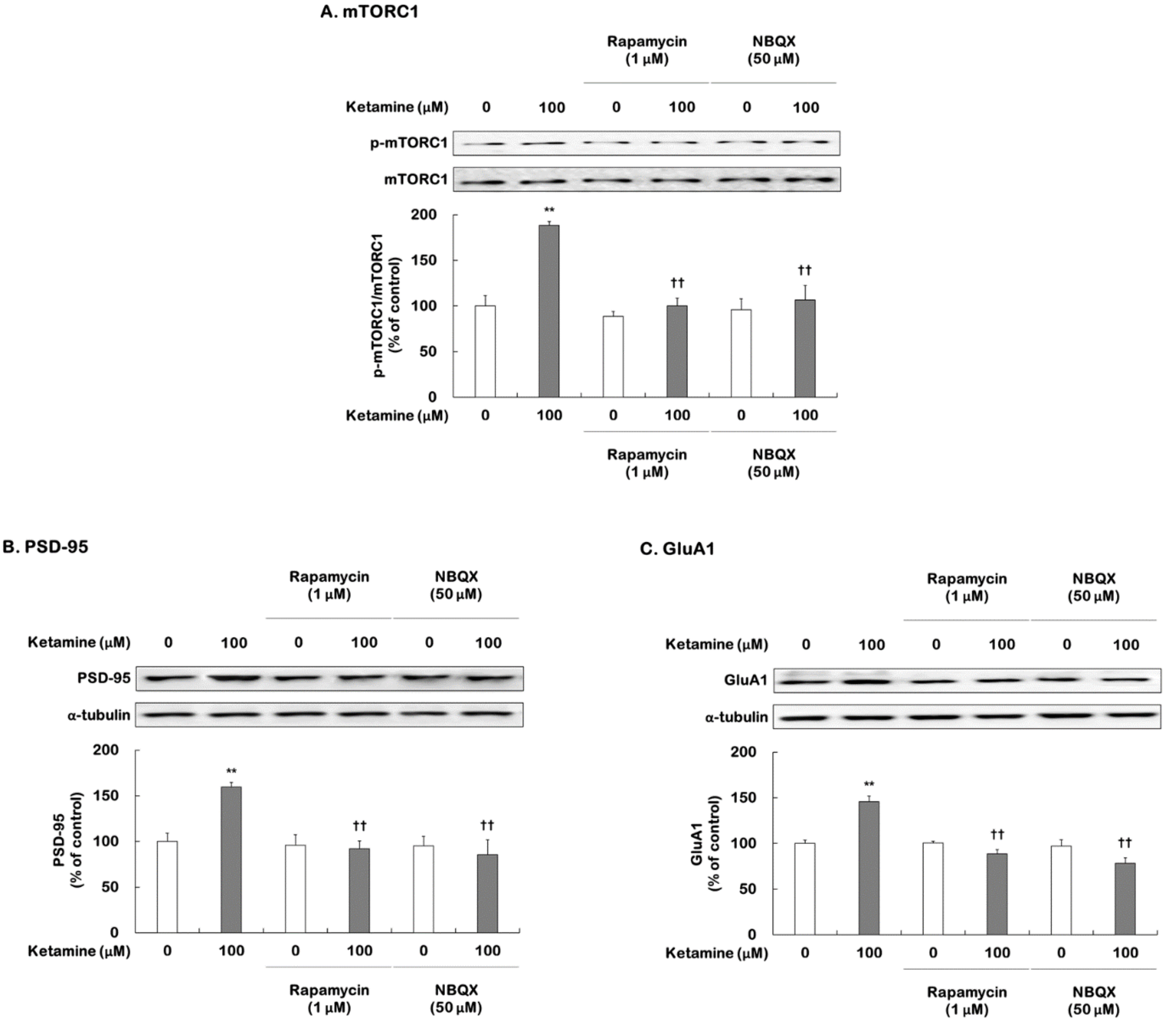
A. mTORC1
Rapamycin (1 μM)
NBQX (50 μM)
Ketamine (μM) 0 100 0 100 0 100
p-mTORC1
mTORC1
p-mTORC1/mTORC1 (% of control)
200
150
100
50
0
**
††
††
B. PSD-95
PSD-95
α-tubulin
PSD-95 (% of control)
C. GluA1
GluA1
α-tubulin
GluA1 (% of control)

Figure S2. Effects of rapamycin or NBQX on increases in mTORC1 phosphorylation, PSD-95, and GluA1 levels induced by ketamine in DEX-treated hippocampal cells.

Cells were exposed to rapamycin (1 μM, mTORC1 inhibitor) or NBQX (50 μM, AMPA receptor inhibitor) for 30 min prior to adding ketamine (100 μM) or DMSO (non-drug treatment control, final concentration 0.5%) for 4 days with DEX (500 μM). The levels of phospho-Ser^{2448}-mTORC1(A), PSD-95 (B), and GluA1 (C) were measured using Western blotting. The picture is cropped to eliminate samples of cells from experimental groups not included in this publication. The full picture is provided in the Supplementary file. Values represent the mean ± SEM expressed as a percentage of the value for cells treated with DMSO alone (control cells). $^{**}P < 0.01$ vs. cells treated with DMSO alone; $^{\dagger\dagger}P < 0.01$ vs. cells treated with LY31495 alone.

A.

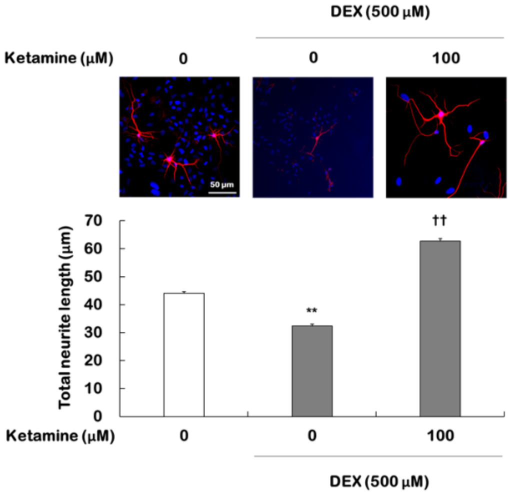

B. Rapamycin

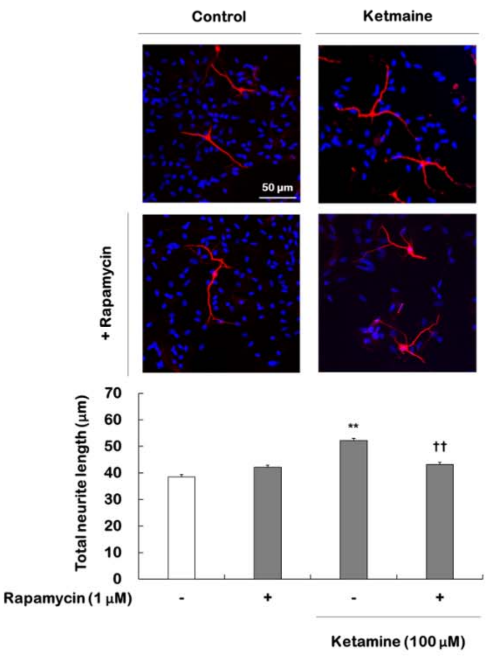

C. NBQX

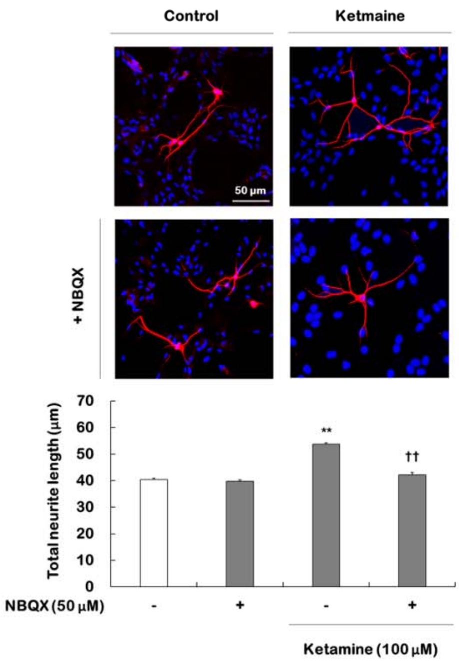

Figure S3. Effects of ketamine on total dendritic length in DEX-treated hippocampal cells: requirement for mTORC1 signaling and AMPA receptor activation.

Cells were treated with ketamine (100 μM) or DMSO (non-drug treatment control, final concentration 0.5%) for 5 days with or without DEX (500 μM) (A). Cells were exposed to rapamycin (1 μM, B) or NBQX (50 μM, C) for 30 min prior to adding ketamine (100 μM) or DMSO for 5 days with DEX. A total of 400 cells from each group were analyzed. All data are expressed as the mean ± SEM. A) $^{**}P < 0.01$ vs. DMSO-treated, non-DEX-treated cells (control cells); $^{\dagger\dagger}P < 0.01$ vs. DMSO-treated, DEX-treated cells; B and C) $^{**}P < 0.01$ vs. cells treated with DMSO alone (control cells); $^{\dagger\dagger}P < 0.01$ vs. LY31495 alone-treated cells.

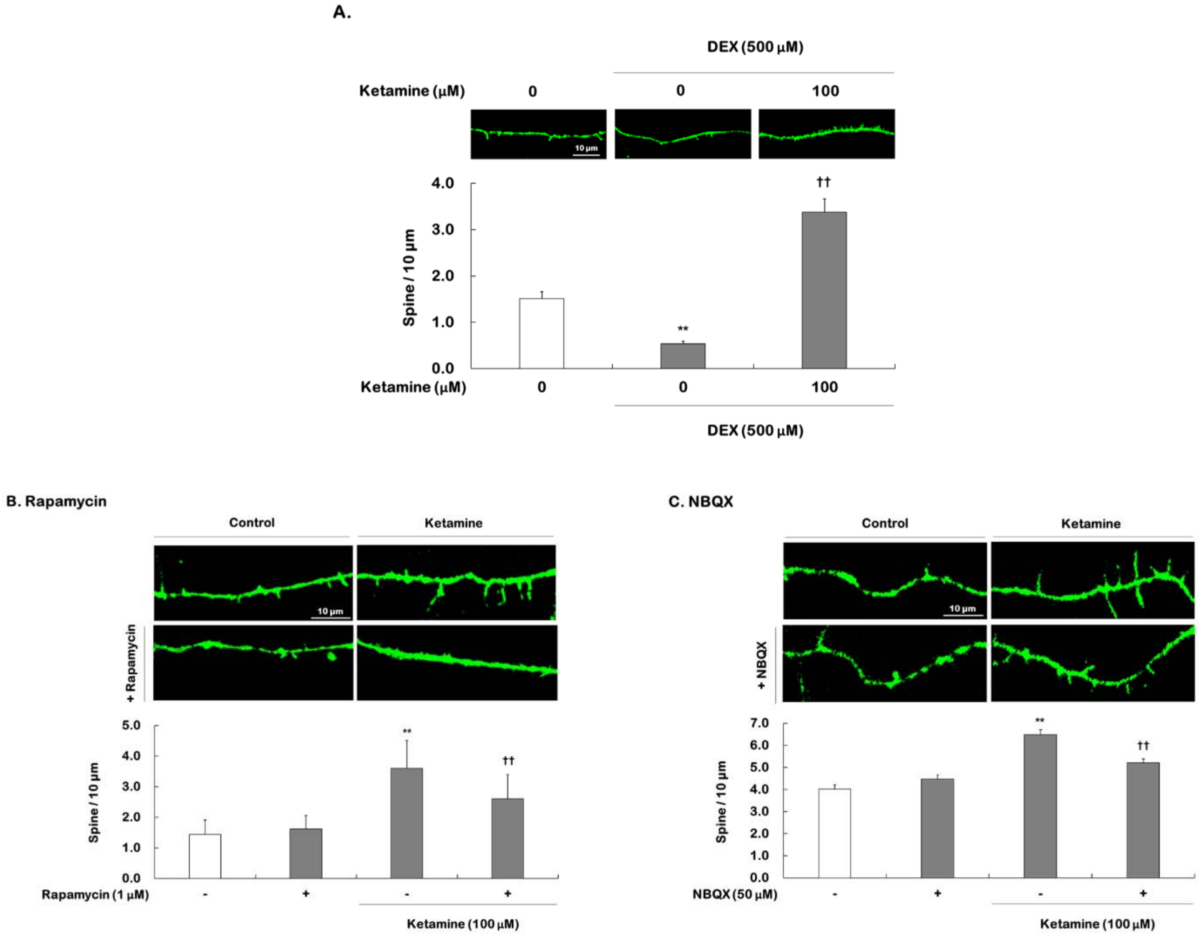

Figure S4. Effects of ketamine on spine density in DEX-treated hippocampal cells: requirement for mTORC1 signaling and AMPA receptor activation.

Cells were treated with ketamine (100 μM) or DMSO (non-drug treatment control, final concentration 0.5%) for 5 days with or without DEX (500 μM) (A). Cells were exposed to rapamycin (1 μM, B) or NBQX (50 μM, C) for 30 min prior to adding ketamine (100 μM) or DMSO for 5 days with DEX. A total of 50 to 60 dendritic segments from each group were analyzed. All data are expressed as the mean ± SEM. A) $^{**}P < 0.01$ vs. DMSO-treated, non-DEX-treated cells (control cells); $^{††}P < 0.01$ vs. DMSO-treated, DEX-treated cells; B and C) $^{**}P < 0.01$ vs. cells treated with DMSO alone (control cells); $^{††}P < 0.01$ vs. LY31495 alone-treated cells.

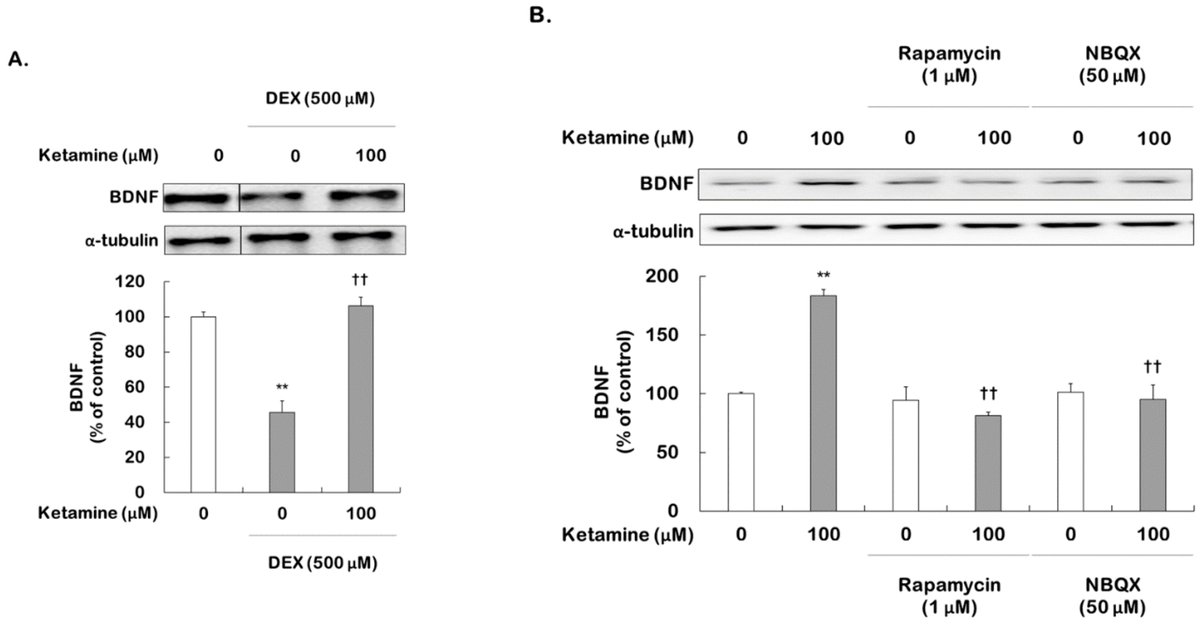

Figure S5. Effects of ketamine on BDNF expression in DEX-treated hippocampal cells: requirement for mTORC1 signaling and AMPA receptor activation.

(A) Cells were treated with ketamine (100 μM) or DMSO (non-drug treatment control, final concentration 0.5%) for 4 days with or without DEX (500 μM). The levels of BDNF expression were measured using Western blotting. The picture is cropped to eliminate samples of cells from experimental groups not included in this publication. The full picture is provided in the Supplementary file. Values represent the mean ± SEM expressed as a percentage of the value for DMSO-treated, non-DEX-treated cells (control cells). $^{*}P < 0.01$ vs. DMSO-treated, non-DEX-treated cells; $^{\dagger}P < 0.01$ vs. DMSO-treated, DEX-treated cells. (B) Cells were exposed to rapamycin (1 μM, mTORC1 inhibitor) or NBQX (50 μM, AMPA receptor inhibitor) for 30 min prior to adding ketamine (100 μM) or for 4 days with DEX. Values represent the mean ± SEM expressed as a percentage of the value for cells treated with DMSO alone (control cells). $^{**}P <$ 0.01 vs. cells treated with DMSO alone; $^{\dagger\dagger}P < 0.01$ vs. ketamine alone-treated cells.

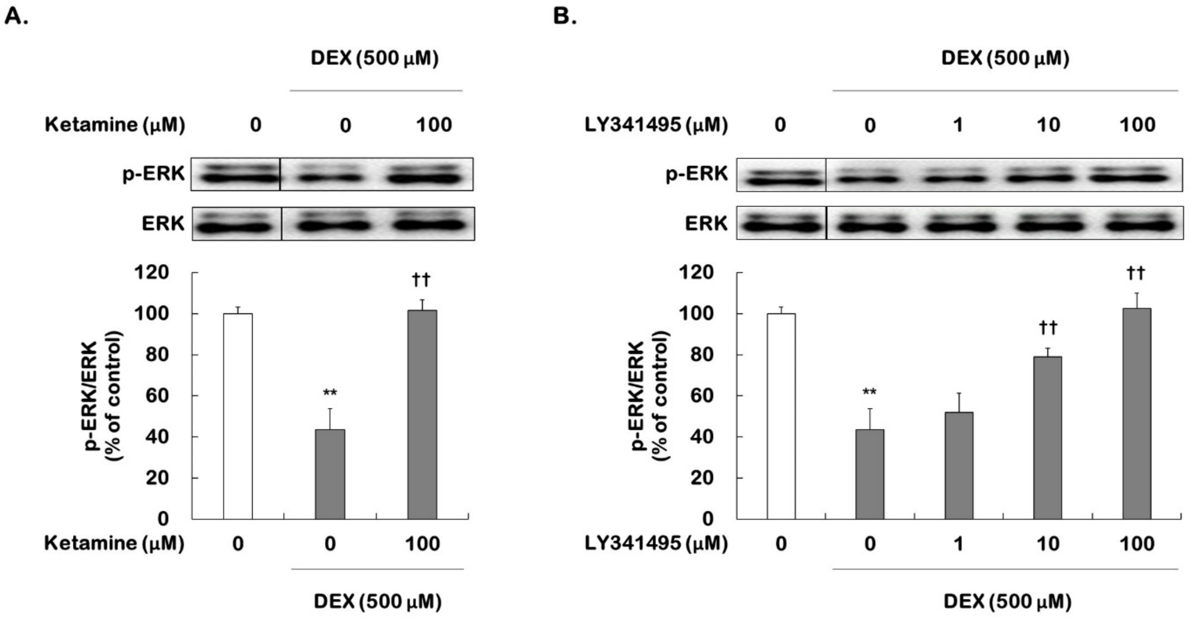

Figure S6. Effects of ketamine and LY341495 on the phosphorylation levels of ERK expression in DEX-treated hippocampal cells.

Cells were treated with ketamine (100 μM) (A), LY341495 (1, 10 and 100 μM) (B), or DMSO (non-drug treatment control, final concentration 0.5%) for 4 days with or without DEX (500 μM). The levels of phospho-Thr^{202}/Tyr^{204}-ERK were measured via Western blotting. The picture is cropped to eliminate samples of cells from experimental groups not included in this publication. The full picture is provided in the Supplementary file. Values represent the mean ± SEM expressed as a percentage of the value for DMSO-treated, non-DEX-treated cells (control cells). $^{**}P < 0.01$ vs. DMSO-treated, non-DEX-treated cells; $^{\dagger\dagger}P < 0.01$ vs. DMSO-treated, DEX-treated cells.

Crude gels from Figure 1.

A.

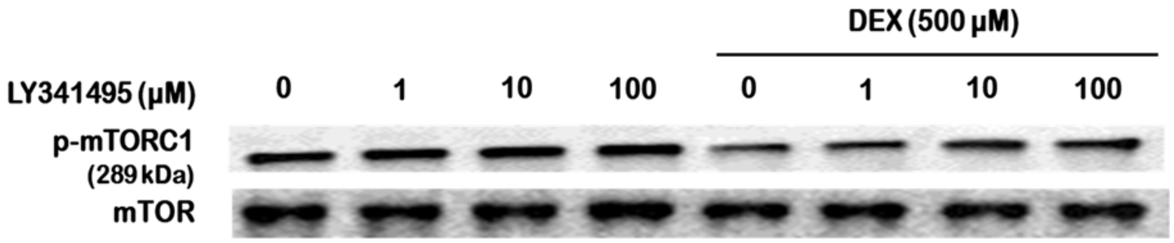

B.

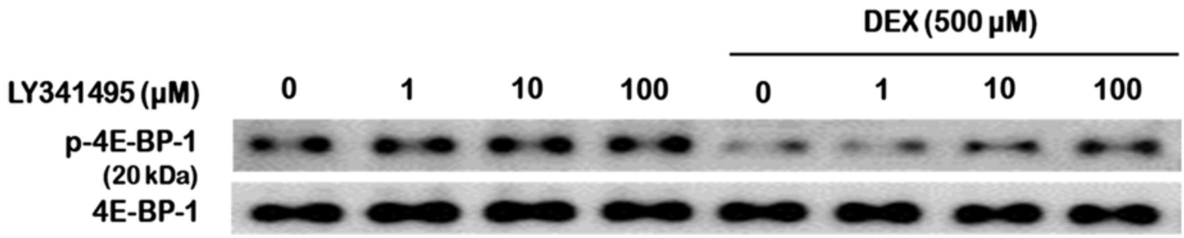

C.

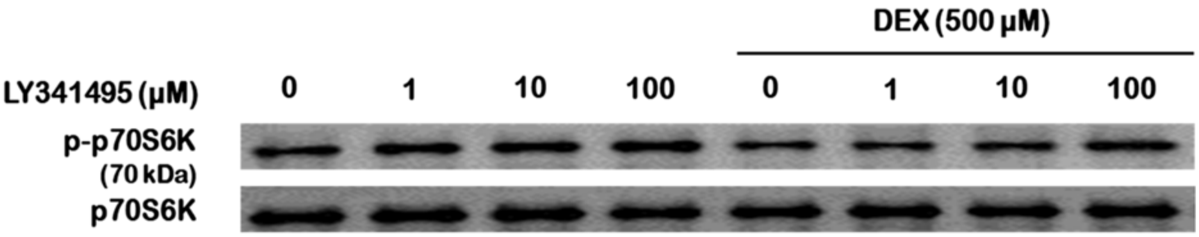

D.

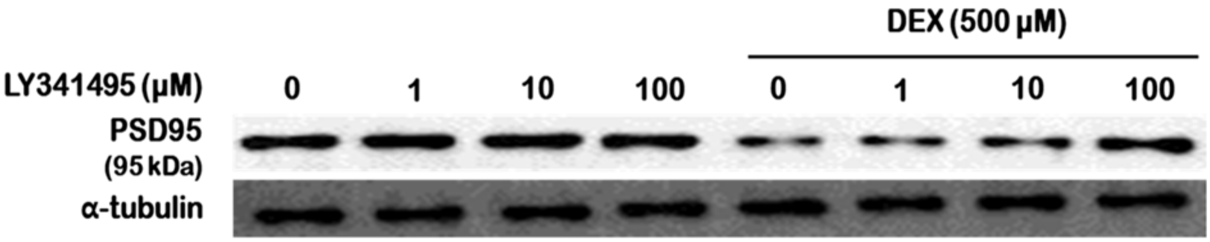

E.

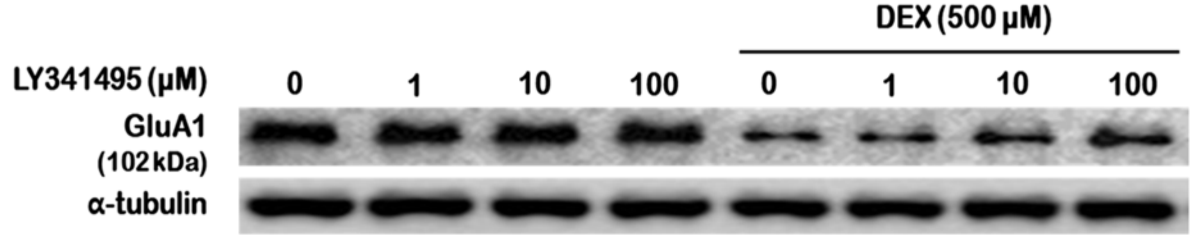

The original images from which we constructed figure 1 A-E (see it below) are shown. Some wells were excluded because the corresponding experimental groups (LY341495 without DEX) are not treated in this research paper.

A. mTORC1

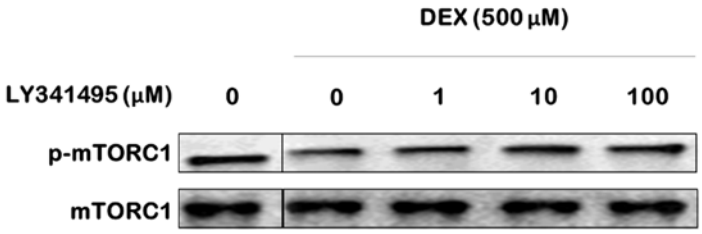

B. 4E-BP-1

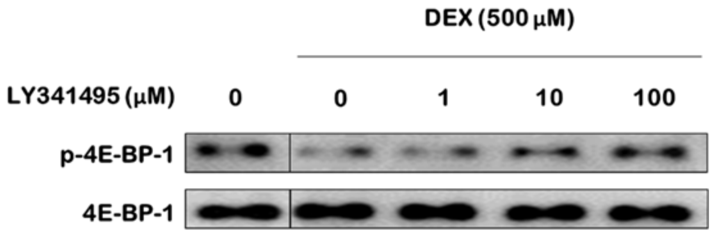

C. p70S6K

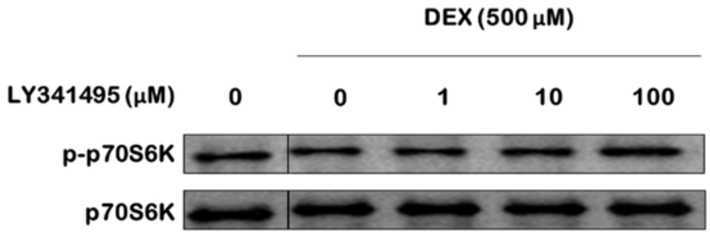

D. PSD-95

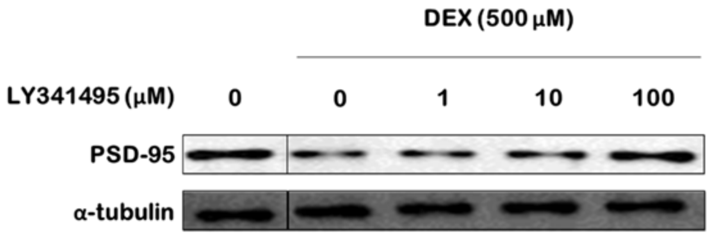

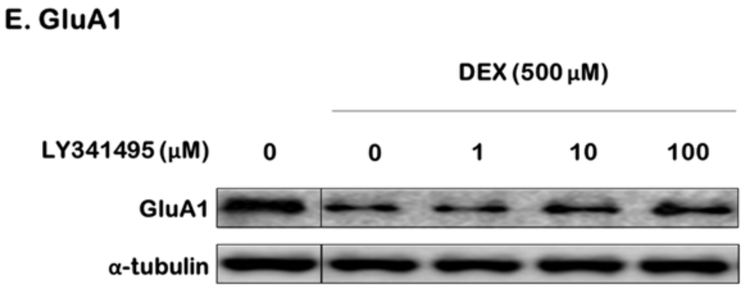

Figure 1. Effects of LY341495 on the levels of mTORC1, 4E-BP1, and p70S6K phosphorylation and PSD-95 and GluA1 expression in DEX-treated hippocampal cells.

Crude gels from Figure 2.

A.

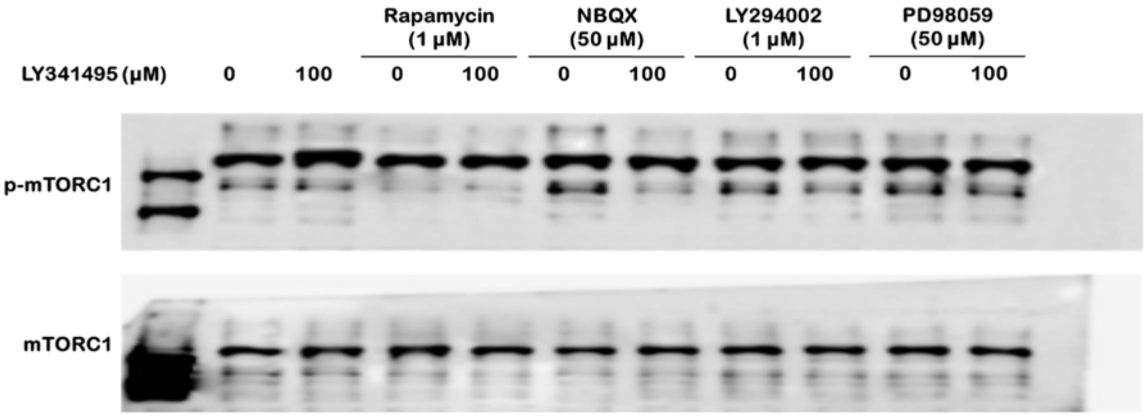

B.

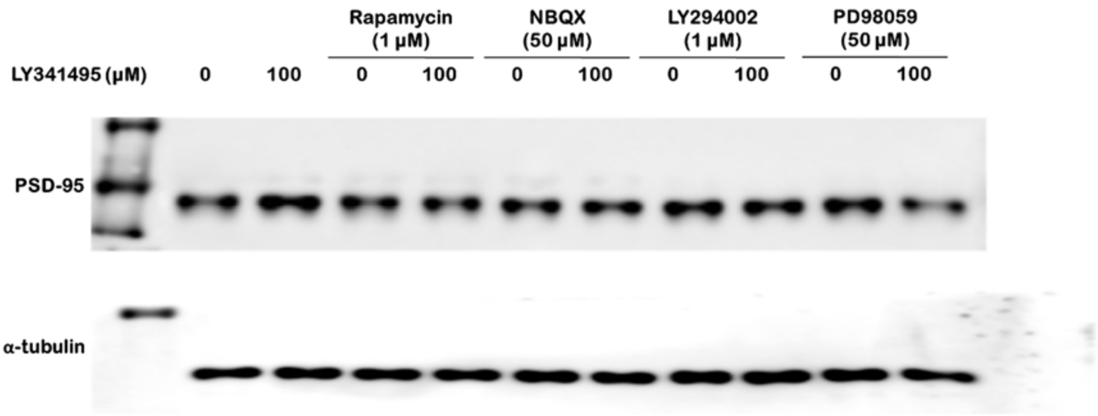

C.

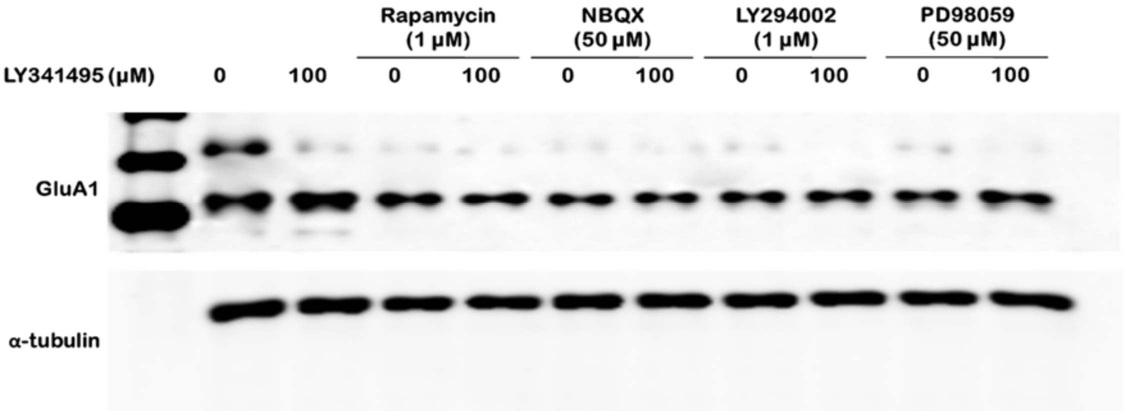

The original images from which we constructed figure 2 A-C (see it below) are shown. Some wells were excluded because the corresponding experimental groups (LY341495 with LY294002 or PD98059) are not treated in this research paper.

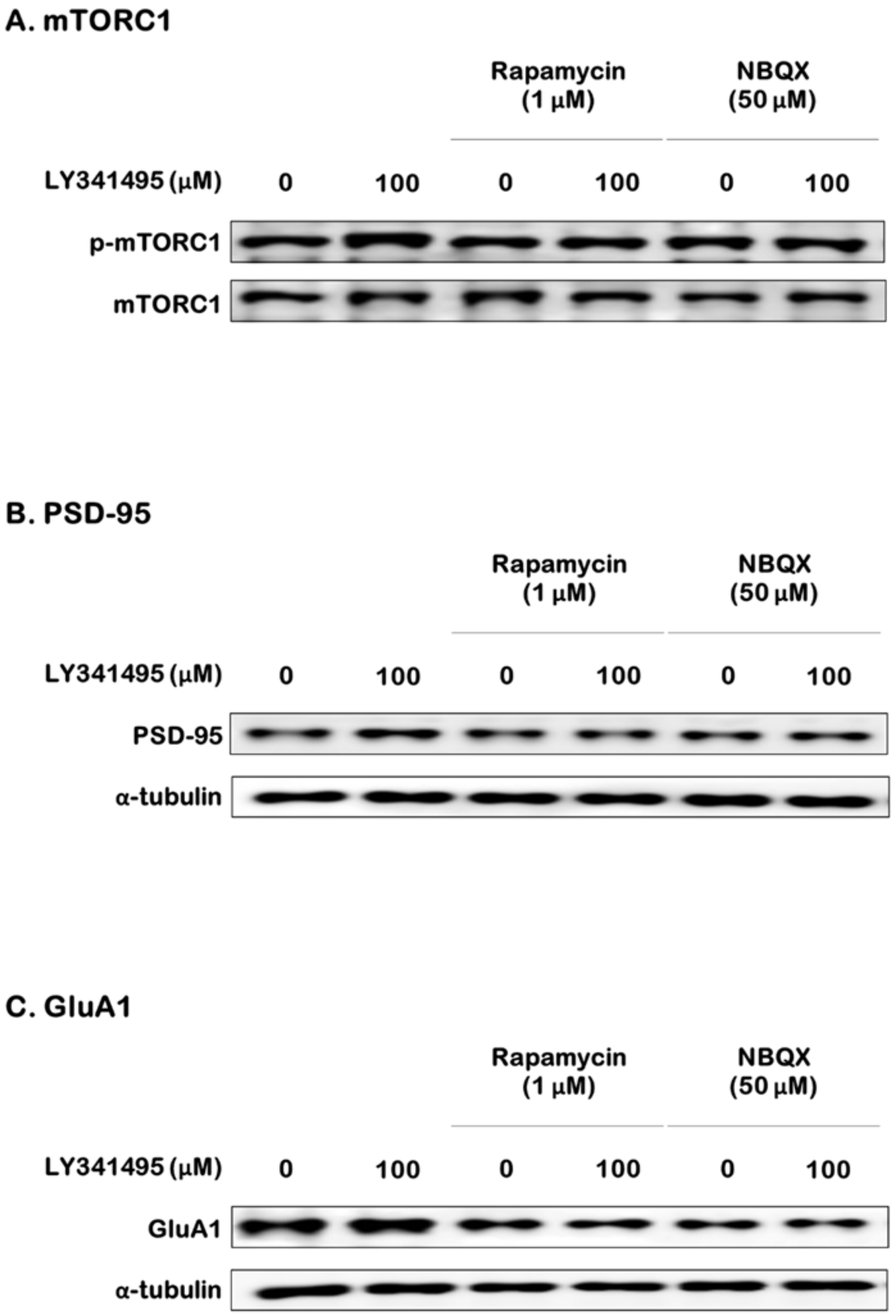

Figure 2. Effects of rapamycin or NBQX on increases in mTORC1 phosphorylation, PSD-95, and GluA1 levels induced by LY341495 in DEX-treated hippocampal cells.

Crude gels from Figure 5.

A.

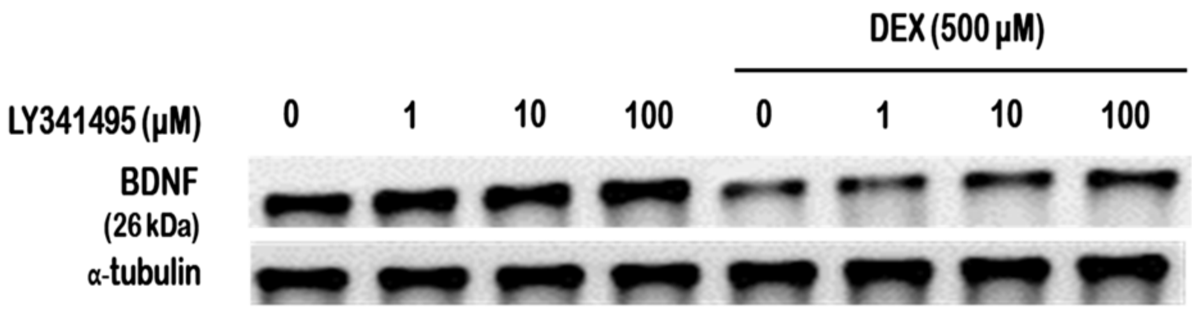

B.

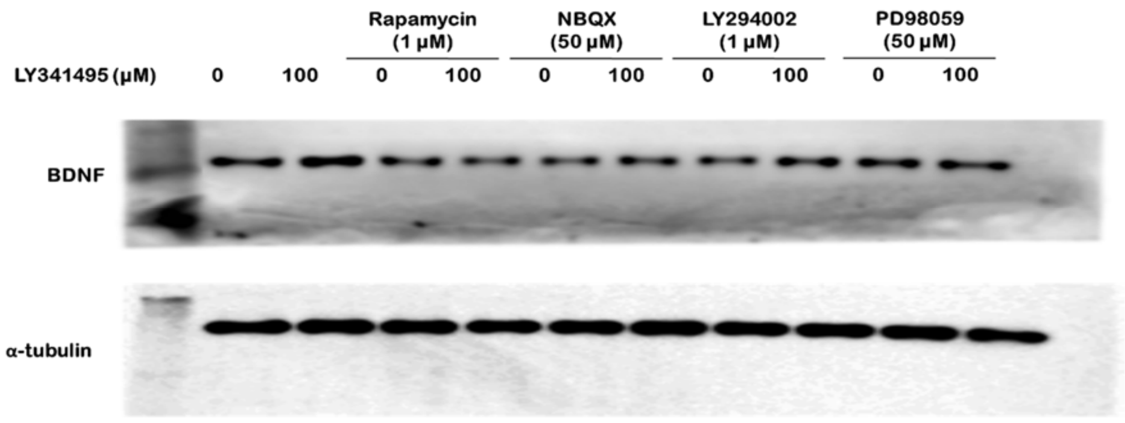

The original images from which we constructed figure 5 A-B (see it below) are shown. Some wells were excluded because the corresponding experimental groups (A; LY341495 without DEX, B; LY341495 with LY294002 or PD98059) are not treated in this research paper.

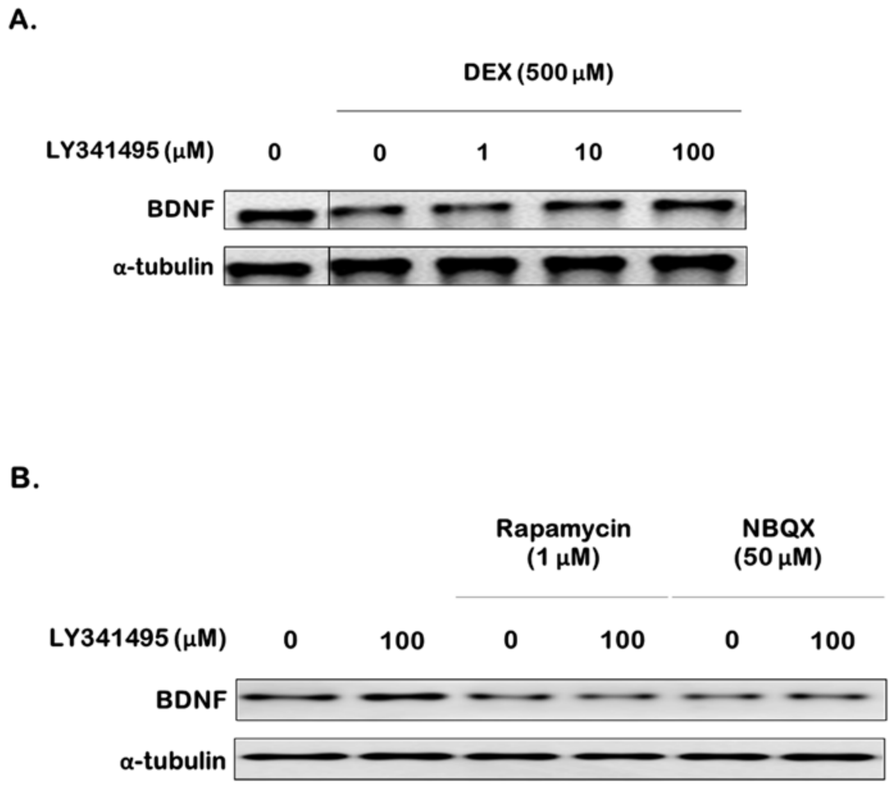

Figure 5. Effects of LY341495 on BDNF expression in DEX-treated hippocampal cells: requirement for mTORC1 signaling and AMPA receptor activation

Crude gels from Figure S1.

A.

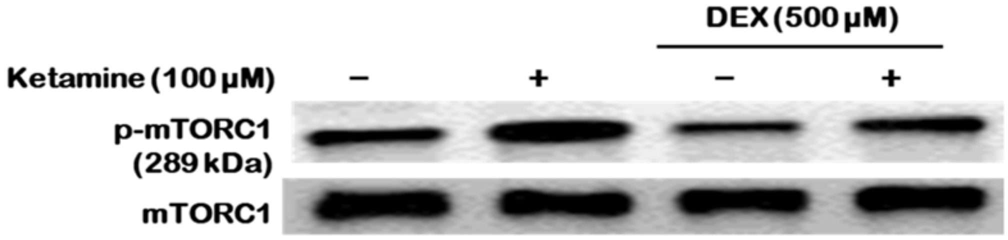

B.

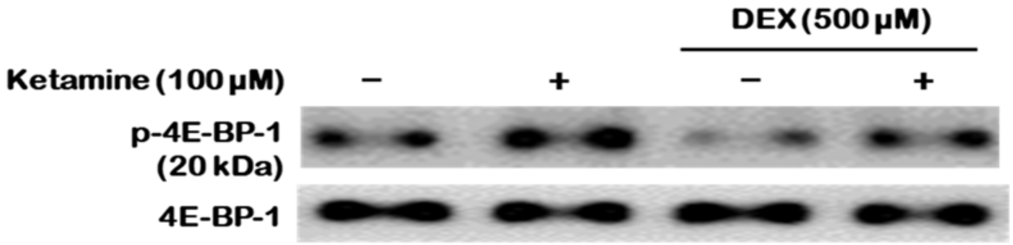

C.

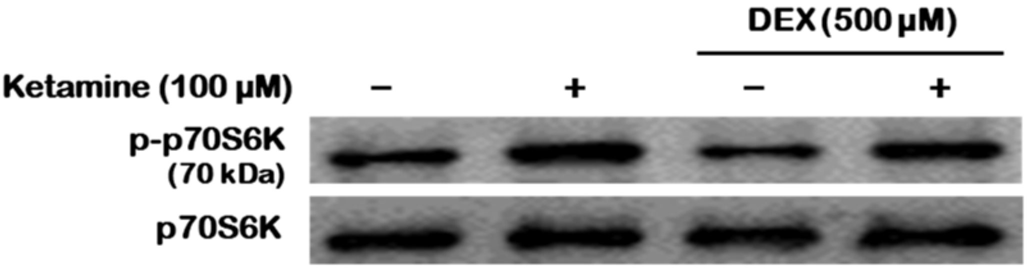

D.

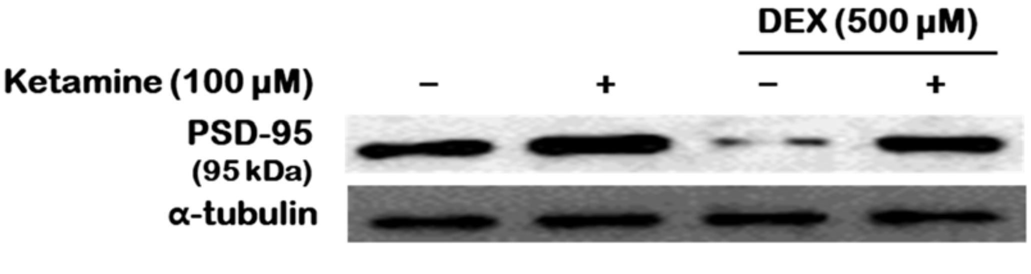

E.

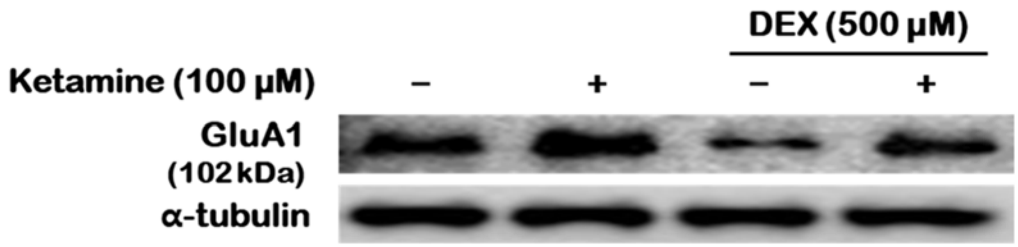

The original images from which we constructed figure S1 A-E (see it below) are shown. Some wells were excluded because the corresponding experimental groups (ketamine without DEX) are not treated in this research paper.

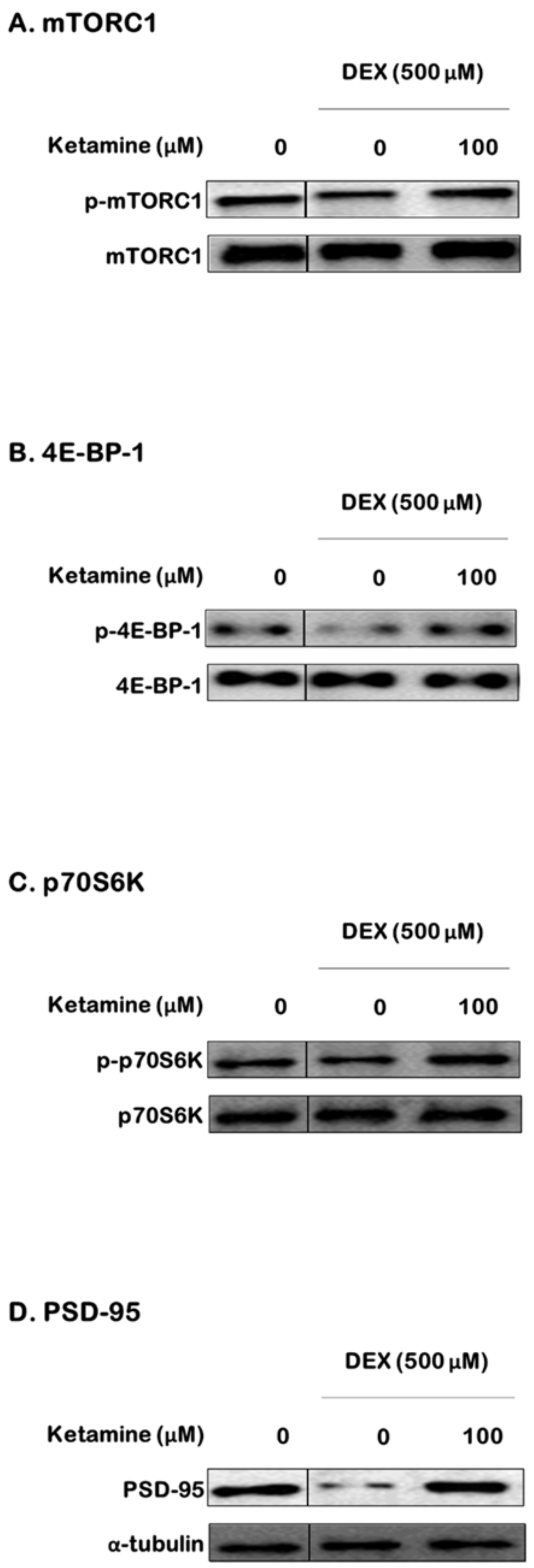
A. mTORC1
DEX (500 μM)
Ketamine (μM) 0 0 100
p-mTORC1
mTORC1
B. 4E-BP-1
DEX (500 μM)
Ketamine (μM) 0 0 100
p-4E-BP-1
4E-BP-1
C. p70S6K
DEX (500 μM)
Ketamine (μM) 0 0 100
p-p70S6K
p70S6K
D. PSD-95
DEX (500 μM)
Ketamine (μM) 0 0 100
PSD-95
α-tubulin

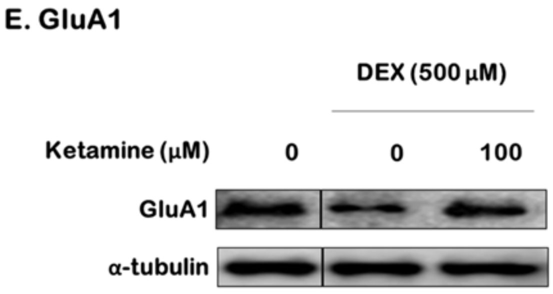

Figure S1. Effects of ketamine on the phosphorylation levels of mTORC1, 4E-BP1, and p70S6K and PSD-95 and GluA1 expression in DEX-treated hippocampal cells.

Crude gels from Figure S2.

A.

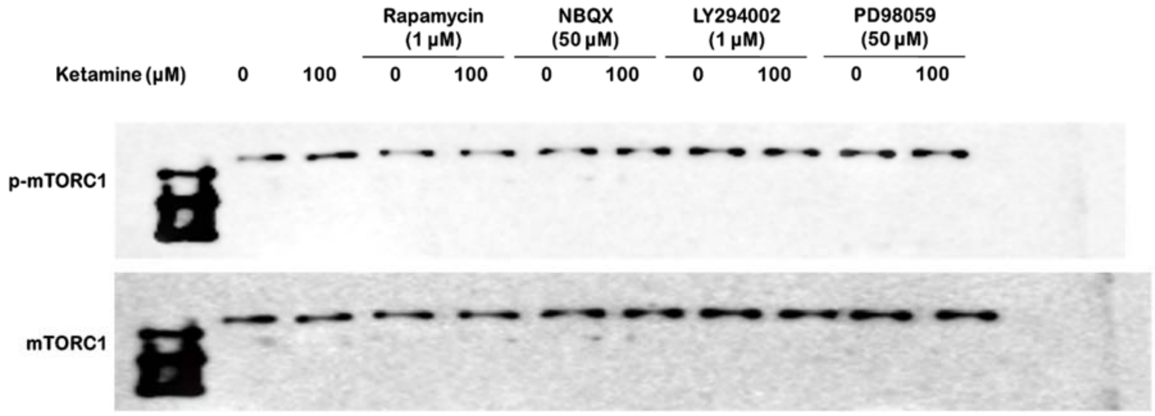

B.

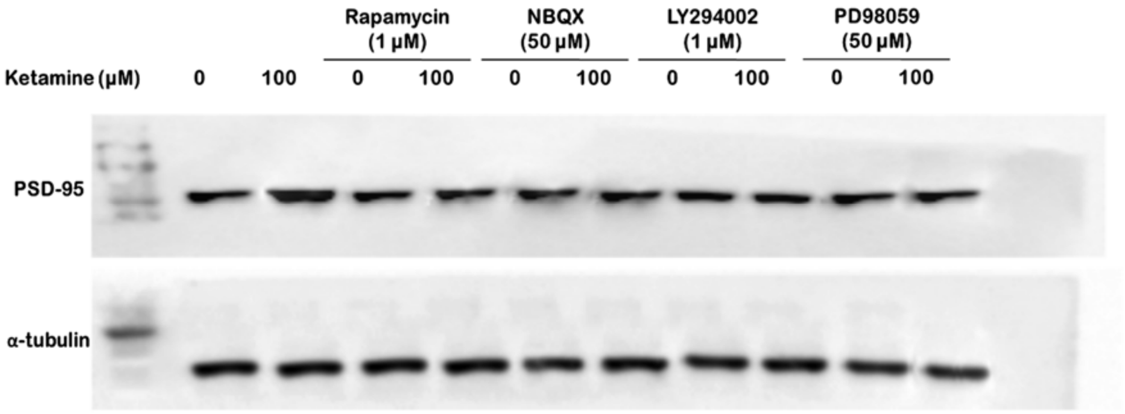

C.

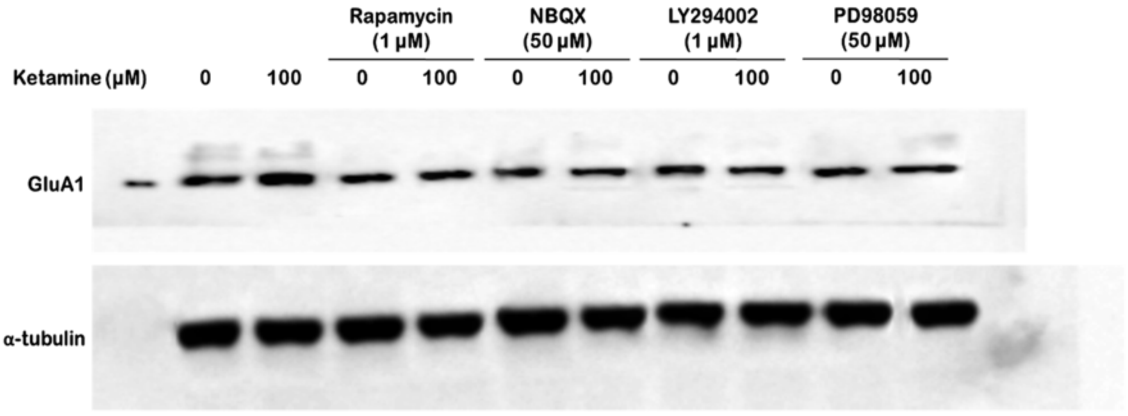

The original images from which we constructed figure S2 A-C (see it below) are shown. Some wells were excluded because the corresponding experimental groups (ketamine with LY294002 or PD98059) are not treated in this research paper.

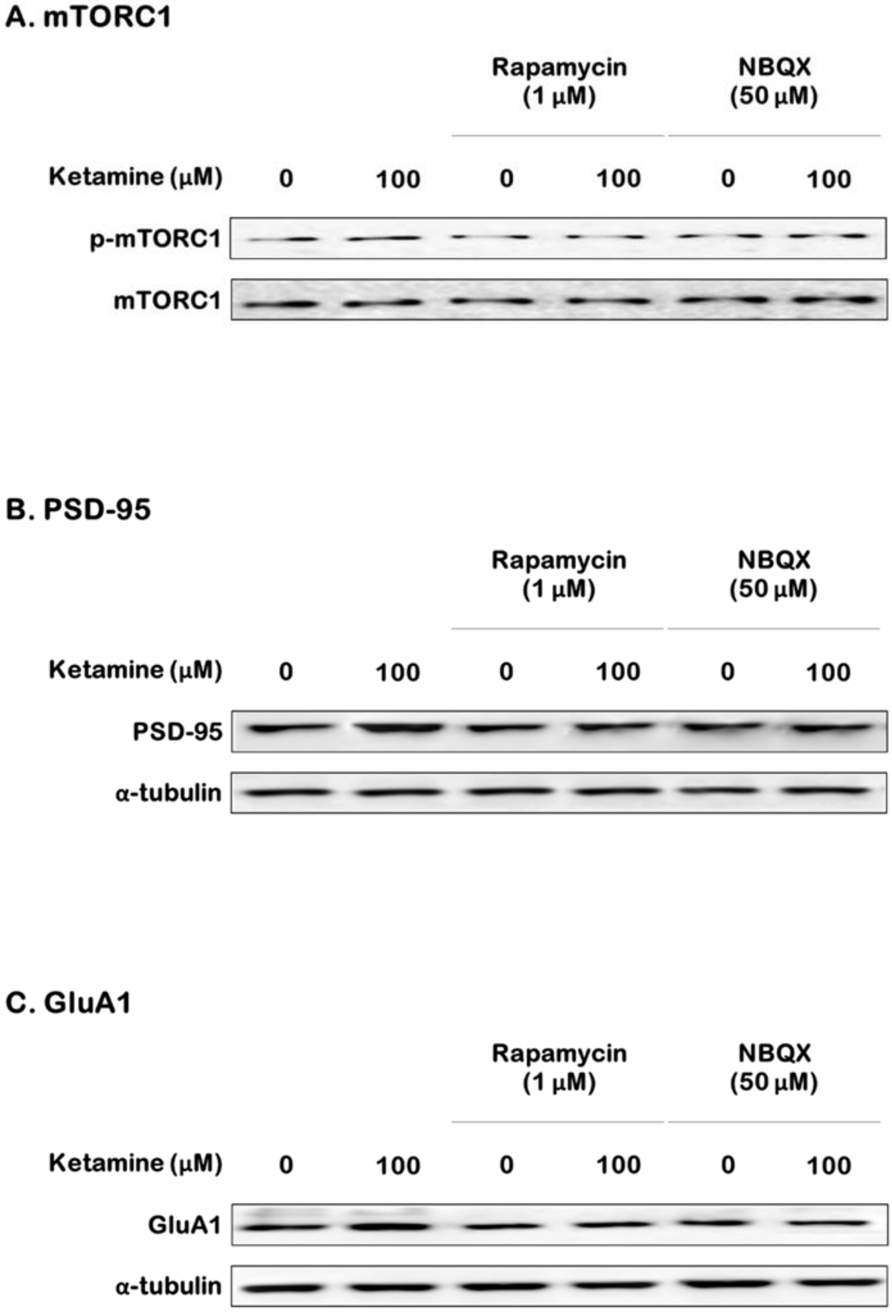

Figure S2. Effects of rapamycin or NBQX on increases in mTORC1 phosphorylation, PSD-95, and GluA1 levels induced by ketamine in DEX-treated hippocampal cells.

Crude gels from Figure S3.

A.

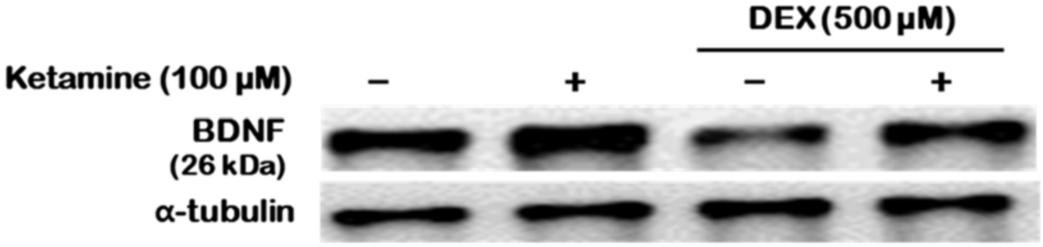

B.

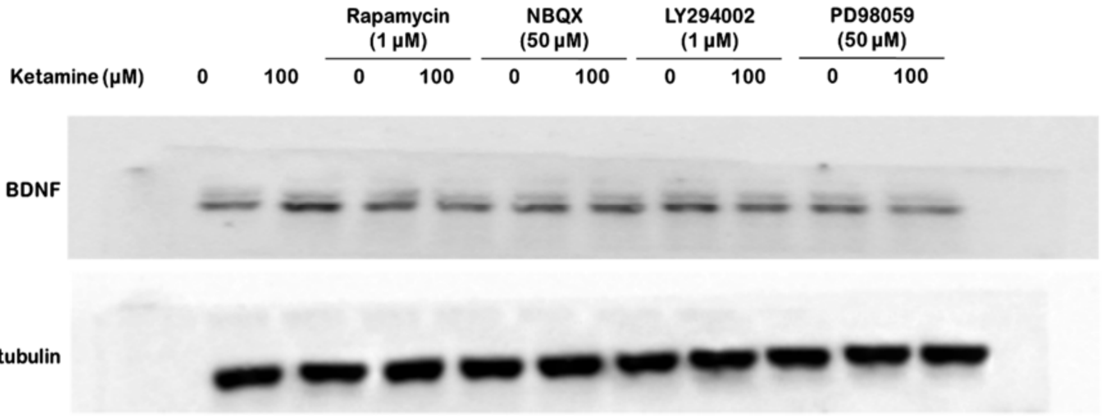

The original images from which we constructed figure S3 A-B (see it below) are shown. Some wells were excluded because the corresponding experimental groups (A; ketamine without DEX, B; Ketamine with LY294002 or PD98059) are not treated in this research paper.

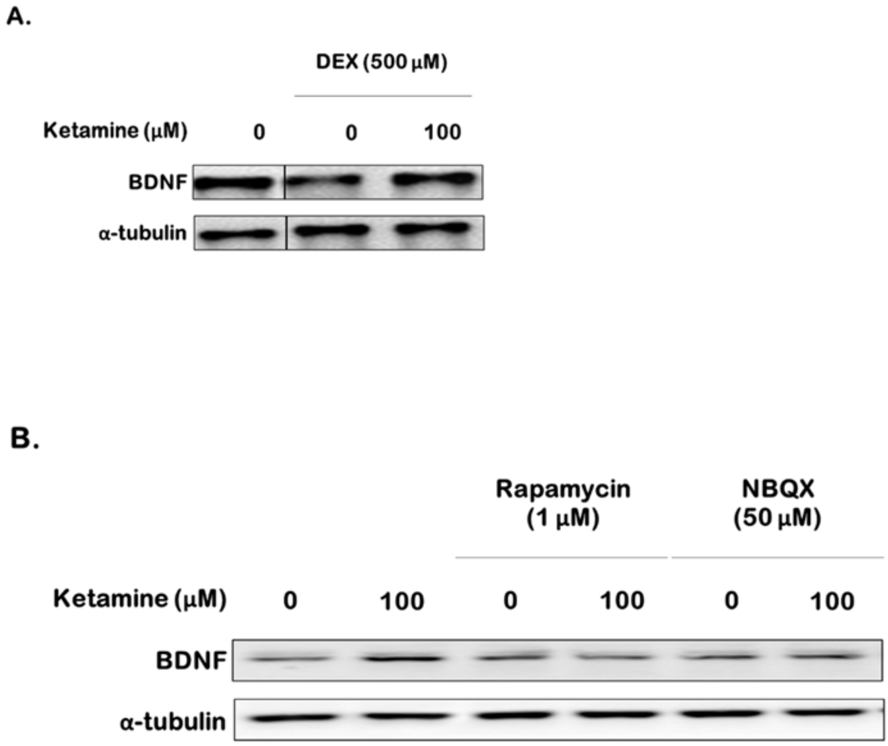

Figure S3. Effects of ketamine on BDNF expression in DEX-treated hippocampal cells: requirement for mTORC1 signaling and AMPA receptor activation.

Crude gels from Figure S6.

A.

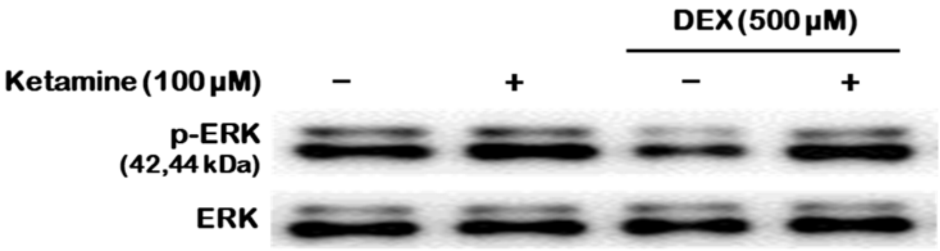

B.

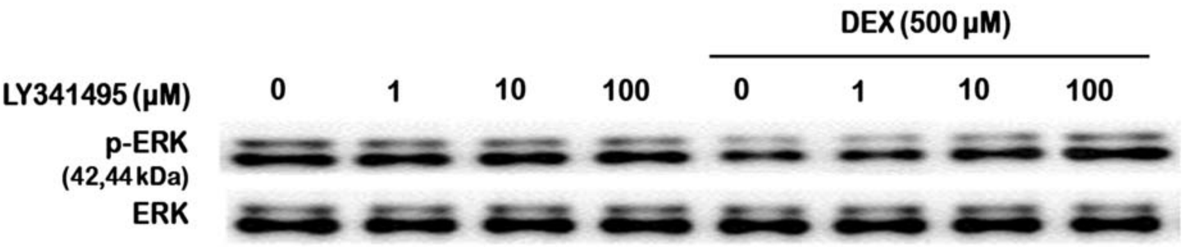

The original images from which we constructed figure S6 A-B (see it below) are shown. Some wells were excluded because the corresponding experimental (A; ketamine without DEX, B; LY341495 without DEX) are not treated in this research paper.

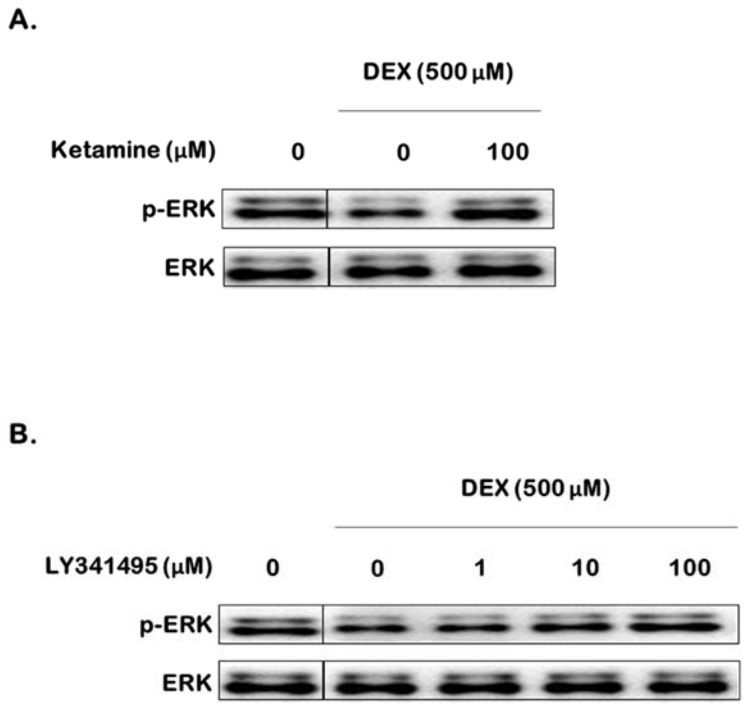

Figure S6. Effects of ketamine and LY341495 on the phosphorylation levels of ERK expression in DEX-treated hippocampal cells**.**
